# Ancient lineages of the keratin-associated protein (KRTAP) genes and their co-option in the evolution of the hair follicle

**DOI:** 10.1186/s12862-023-02107-z

**Published:** 2023-03-20

**Authors:** Thomas Litman, Wilfred D. Stein

**Affiliations:** 1 Department of Immunology and Microbiology, University of Copenhagen, Mærsk Tower 07-12-70 Nørre Allé 14, 2200 Copenhagen N, Denmark; 2 Silberman Institute of Life Sciences, Hebrew University, 91904 Jerusalem, Israel

**Keywords:** Hair, Evolution, Placodes, Keratin-associated proteins, Keratins, Trichocyte

## Abstract

BLAST searches against the human genome showed that of the 93 keratin-associated proteins (KRTAPs) of *Homo sapiens*, 53 can be linked by sequence similarity to an *H. sapiens* metallothionein and 16 others can be linked similarly to occludin, while the remaining KRTAPs can themselves be linked to one or other of those 69 directly-linked proteins. The metallothionein-linked KRTAPs comprise the high-sulphur and ultrahigh-sulphur KRTAPs and are larger than the occludin-linked set, which includes the tyrosine- and glycine-containing KRTAPs. KRTAPs linked to metallothionein appeared in increasing numbers as evolution advanced from the deuterostomia, where KRTAP-like proteins with strong sequence similarity to their mammalian congeners were found in a sea anemone and a starfish. Those linked to occludins arose only with the later-evolved mollusca, where a KRTAP homologous with its mammalian congener was found in snails. The presence of antecedents of the mammalian KRTAPs in a starfish, a sea anemone, snails, fish, amphibia, reptiles and birds, all of them animals that lack hair, suggests that some KRTAPs may have a physiological role beyond that of determining the characteristics of hair fibres. We suggest that homologues of these KRTAPs found in non-hairy animals were co-opted by placodes, formed by the ectodysplasin pathway, to produce the first hair-producing cells, the trichocytes of the hair follicles.

**Supplementary Information:**

The online version contains supplementary material available at 10.1186/s12862-023-02107-z.

## Introduction

The human genome contains 93 protein-coding genes that are members of the keratin-associated protein (*KRTAP*) family, concerned with the properties of hair, wool, and fur. These genes are divided among 26 sub-families, closely intra-related, numbered *KRTAP1* through *KRTAP29*. The *KRTAP10, KRTAP4* and *KRTAP5* sub-families are the largest with 11 or 12 members in each. The sub-families are grouped into those encoding proteins having a high or ultra-high sulphur (contributed by cysteine) content, and those with a high content of glycine and tyrosine [1]. As their name implies, the proteins encoded by these genes interact structurally with keratin proteins, this being attested to directly in binding studies using isothermal titration calorimetry [2]. Within the trichocytes (the hair-producing cells), the keratins and the keratin-associated proteins act together to form the hair fibres [3] determining the particular characteristics of the different types of hair, fur, wool and quills found in various animals. Studies on wool, as an important commercial product, have led to the identification of various KRTAP family members (KRTAPs 6–3 and 20–2 in particular) that convey structural properties such as fibre thickness and curliness to the hair fibres [4, 5]. Animals living in different environments have evolved appropriate selections of the KRTAPs [6].

Hair is one of the defining features of the mammals. The remnants of hair fibres identified within coprolites found in Upper Permian strata [7] provide suggestive evidence that hair was present in the most primitive mammals (see also [8]), as does the claim [9] of whiskers being present in therapsids, the mammal-like reptiles of the Early Permian (some 290 million years ago). The presence of hair in such early mammals is thought to be associated with the regulation of an elevated body temperature that would have been fostered by the insulating properties of hair, while the function of the whiskers would have been tactile. Both these features would enable the adoption of the mammals’ ecological niche, a nocturnal life style. Interestingly, hair is involved also in heat dissipation. Heat dissipation is a major problem for elephants whose large bulk is associated with a high production of heat. The almost invisible fine hairs on the skin of elephants has been shown to be responsible for up to 23% of the heat dissipation in these animals at low wind speeds, where their thermoregulation needs are greatest [10]. That hair was preceded in evolution by spines was suggested by Alibardi and Rogers [11].

Writing on the evolutionary sources of the KRTAP-encoding genes, Wu et al. [1] reported that the keratin-associated proteins were confined to the mammals, while in a later paper, they wrote: “As the sequence composition of the *KRTAP* genes have no homology with other existing genes, it is likely that the *KRTAP* genes originated de novo from non-genic regions.” Wu and Irwin [12]. However, Alibardi et al. [13] had already, on the basis of sequence similarities, pointed to possible evolutionary relationships between keratins of the lizard, the lamprey and even the lancelet and the keratin-associated proteins of the goat, mouse and humans. The present paper is an attempt to dig deeper into this issue, taking into account the many recent additions to the genomic databases. We find proteins annotated as KRTAPs or KRTAP-like proteins in animals going as far back in evolution as the snail, and even the sea anemone and starfish. In addition, we find that there is significant sequence similarity between KRTAP proteins and proteins that originated well before the mammalian KRTAPs, namely, occludin and the metallothioneins. We suggest that these similarities might point to occludin and the metallothioneins as being close to the original sources of the KRTAPs. Consistent with our finding of KRTAP proteins in animals that arose long before the evolution of hair, we point to published evidence suggesting that certain KRTAP proteins have a function in driving cell proliferation, this being in addition to their function in controlling the characteristics of hair.

## Methods and definitions

Searches for similarity among the KRTAPs themselves and between other proteins were performed using the protein BLAST (Basic Local Alignment Search Tool) program of the NCBI (National Center for Biotechnology Information of the National Library of Medicine): https://blast.ncbi.nlm.nih.gov/Blast.cgi with the following search parameters: Max target sequences 1000, Expect threshold 200,000, Word size 2, Max matches in a query range 0, Matrix BLOSUM62, Gap Costs Existence: 11 Extension: 1, No compositional adjustments, No Low complexity regions filter.

In general, we collected the top 500 closest matches, defining these as proteins that are **linked to** the bait used in the BLAST search. Proteins with Expect values in the BLAST search lower than or equal to 10^–3^ compared with the bait were defined as **similar in sequence** to the bait. Proteins that were annotated as KRTAP proteins or as KRTAP-like proteins were selected from the top 500 matches and listed as the KRTAP-annotated harvest of that search. Sequence comparisons between pairs of proteins and dot plots of the comparisons were made using the BLAST 2-sequences tool of the BLAST program, using the same search parameters as above. This procedure was used also to check the closeness of sequence between harvested proteins annotated as KRTAPs or as KRTAP-like and their putative homologues. All one-on-one Expect values recorded in this paper were the results of a BLAST 2-sequences comparison between the two proteins stated.

Alignments between protein sequences were established and dot plots generated using the COBALT (Constraint-Based Alignment Tool) aligner at the NCBI https://www.ncbi.nlm.nih.gov/tools/cobalt/cobalt.cgi?CMD=Web.

To assess *KRTAP* gene expression, we queried the GEO (Gene Expression Omnibus, https://www.ncbi.nlm.nih.gov/geo/) database, from which human skin transcriptome data (accession GSE121212) as well as expression data from Danio rerio (BioProject: PRJEB1986) were downloaded. For the Sea anemone, the Nematostella-specific gene expression database NvERTx (http://nvertx.kahikai.org) developed by Warner et al. was accessed [14].

Tissue specific *KRTAP* expression information was pulled from Uniprot (www.uniprot.org) as recorded in Additional file 3: Table S1.

Ages at which genes were accreted to the evolving human genome were taken from Table 3 of Litman and Stein [15]. The definitions of the phylostratum levels (1 through 19) can be found in Additional file 4: Table S4 “List of the 19 Phylostrata”.

Properties of the KRTAPs were taken from the listings in the GeneALaCart program of GeneCards https://genealacart.genecards.org

## Results

**Fig. 1:**
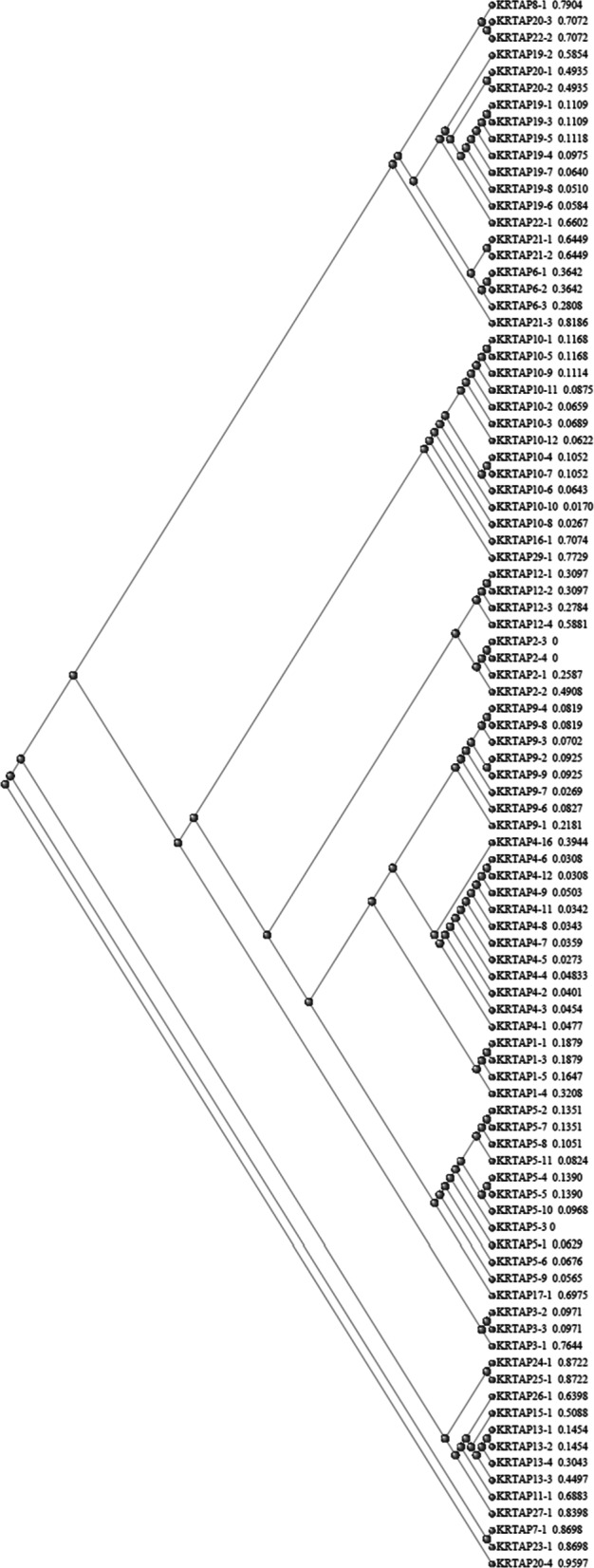
Phylogenetic tree (slanted cladogram) of the 93 KRTAP proteins of *Homo sapiens* generated by applying the COBALT tree method of NCBI. Protein labels are sorted according to distance. as generated by the COBALT program, reported next to the protein symbol

To provide a basis for our study, we built a phylogenetic tree using all the 93 KRTAPs of *Homo sapiens.* The result is depicted as Fig. 1.

It can be seen that there are two main branches of this tree, an upper and a lower branch, with an additional single-membered branch containing KRTAP20-4. In attempts to find possible ancestral proteins for each of the two main branches, we BLASTed members of the upper branch against the human genome and did this also for members of the lower branch. We found that some proteins which were not members of the KRTAP family were frequently found high up in the results of these BLAST searches, mingling with members of the KRTAP family themselves. We reasoned that some of these non-KRTAP proteins might share a common ancestor with the KRTAP proteins. For the proteins in the lower branch, occludin was consistently found in these BLAST searches and was the single protein consistently so found. For the upper branch, however, a number of contenders for the role of ancestral proteins were found (Additional files 1 and 5). Of these, metallothionein appeared in evolution before the mammals and returned the highest number of KRTAPs when BLASTed against the human genome.

Occludin is a 65 kDa integral plasma-membrane protein localized at the tight junctions, while the metallothioneins are a family of cysteine-rich, low molecular weight proteins, localized to the membrane of the Golgi apparatus. The ages at which these two were first accreted to the evolving genome were, for occludin, Phylostratum 12 (the jawless fish) (Table 3 of Litman and Stein 2019) [12], while a metallothionein is already present in the lancelet [16] of phylostratum 9, so both genes appeared early enough to have founders shared with sources for genes appearing in the most primitive mammals, these being found in phylostratum 15. (For an introduction to the concept of phylostratum, see Domazet-Loso and Tautz 2010) [17].

To test whether occludin might be a close source for the KRTAPs, we BLASTed it against the human genome. The result is recorded in worksheet “OCLN vs. Human” in Additional file 1, and shows that many proteins annotated as KRTAPs are indeed related to occludin, being in the top 500 hits of this BLAST search. These proteins are divided amongst the following families: KRTAP19, KRTAP20, KRTAP21, KRTAP6, KRTAP7, and KRTAP8. Eight of these 16 KRTAPs were especially closely related to occludin, having Expect values in the BLAST search of 10^–3^ or less. It will be convenient in what follows to designate these 16 KRTAPs as belonging to the O lineage. All of these proteins were found in the lower branch of the phylogenetic tree of Fig. 1.

We proceeded similarly with the metallothionein MT1A, and the results are recorded in worksheet “MT1A vs. Human” in Additional file 1. We recovered 53 metallothionein-related KRTAP-annotated proteins, divided amongst the following families: KRTAP1, KRTAP3, KRTAP4, KRTAP5, KRTAP9, KRTAP10, KRTAP12, KRTAP17, and KRTAP29. All of these proteins were found in the upper branch of the phylogenetic tree of Fig. 1. Parry and Walters [18], investigating the keratin filaments of the Tuatara *Sphenodon punctatus* had found that the four keratin filament proteins with the highest sulphur content were homologous to the metallothioneins, but the mammalian keratin-associated proteins had no direct equivalent in the Tuatara genome.

### The 53 KRTAPs linked to metallothionein will be designated as the M lineage

None of these 53 KRTAPs that were linked to a metallothionein were in the set of 16 proteins linked to occludin. The combined two sets left 24 KRTAPs of *H. sapiens* that were not directly linked to either occludin or metallothionein, according to the definition of linkage in our "Methods and definitions" section. With each of these 24 proteins as bait, we BLASTed the human genome to see if we could find any other pre-mammalian protein that could be a source for any of these 24, but failed to find a candidate protein. We did, however, find for all of the 24 proteins, links to numerous other KRTAPs that did appear in our listings of proteins linked to occludin or metallothionein. We defined these 24 as being indirectly linked to occludin or to metallothionein. The results of our searches for links to occludin and metallothionein are recorded in column “Source” of Additional file 3: Tsble S1. Note that 6 of the 93 human KRTAPs are indirectly linked both to a directly-linked occludin protein or a directly-linked metallothionein protein. Column “Type” Additional file 3: Tsble S1 o records the results of classifying the 93 proteins into the high sulphur, ultrahigh sulphur or high tyrosine glycine groups using the Aliases section of GeneALaCart (See Methods). Note that all the proteins in the ultra-high sulphur group are in the M lineage, while those in the high sulphur group were either directly linked to metallothionein or (in a few cases) indirectly linked to metallothionein or to both occludin and metallothionein. Fourteen of the KRTAPs in the high tyrosine glycine group are in the O lineage, while the remaining of this group is indirectly linked to occludin.

**Fig. 2:**
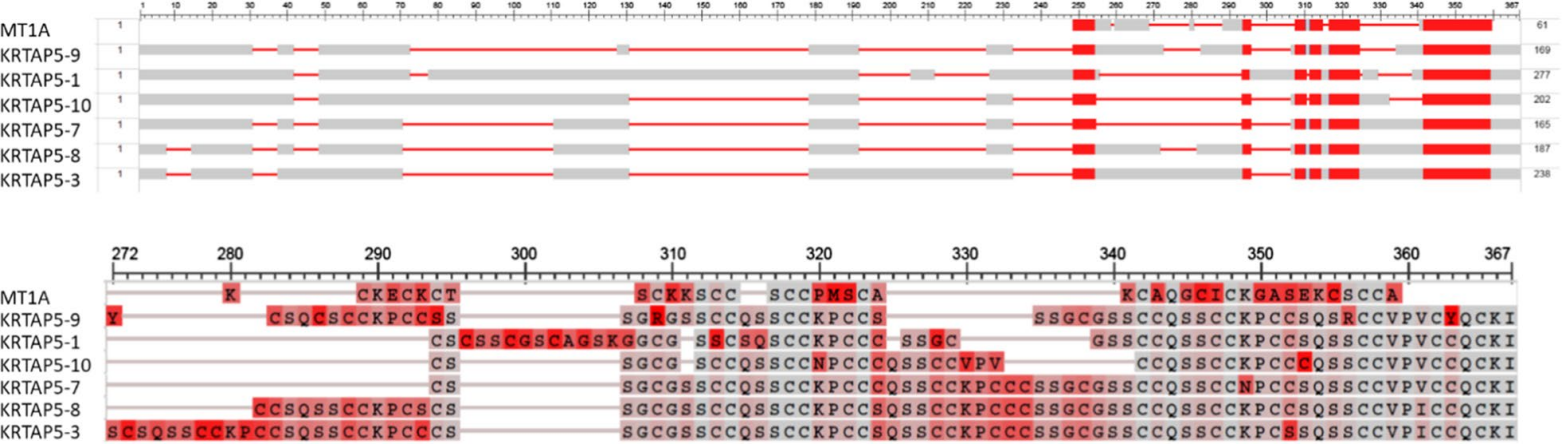
Upper figure: Alignment map comparing the metallothionein (MT1A) used as bait in our BLAST searches (topmost protein, P04731), and a selection of KRTAPs from the metallothionein-linked set. From second line to bottom. KRTAPs 5–9, 5–1, 5–10, 5–7, 5–8, and 5–3. All have Expect values of lower than 5 × 10^–8^ and are thus closely similar to metallothionein MT1A. Lower figure: the highest matched sections on an expanded scale. The colour coding here is the “Column Quality score –Protein”, which assigns scores to residues based on how well a particular residue agrees with the others in the column (grey high to red low). The map was obtained using the COBALT tool at the NCBI

**Fig. 3:**
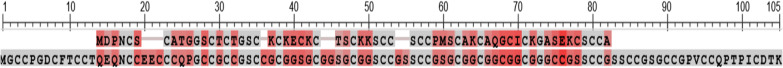
Alignment of the amino-acid sequences of (top line) KRTAP17-1 and (bottom line) metallothionein MT1A. (The colour coding here is the “Column Quality score –Protein”, which assigns scores to residues based on how well a particular residue agrees with the others in the column (grey high to red low). The Expect value for the sequence similarity between these two proteins is 7 × 10^–6^

Figure 2 shows the alignment obtained using COBALT of the comparison between the metallothionein MT1A that we used as our bait (topmost protein) and a selection of KRTAP-annotated proteins from the metallothionein-linked set. The metallothionein aligns with just a portion of the KRTAP proteins, but this is sufficient to reveal strong links to the bait sequence:

Note that 10 of the 20 sulphur-contributing cysteine residues (symbolized by C) in the metallothionein align well with those in these high sulphur KRTAP, although most of the cysteines that the KRTAPs possess are outside the section of the molecule that aligns with the metallothionein. However, in KRTAP17-1, which is one of the smallest of the metallothionein-linked KRTAP genes (105 residues long), 17 of metallothionein’s 20 cysteines match a corresponding 17 of the 37 cysteines of this KRTAP gene and, in addition, there are matches also at 6 other residues (Fig. 3).

**Fig. 4:**
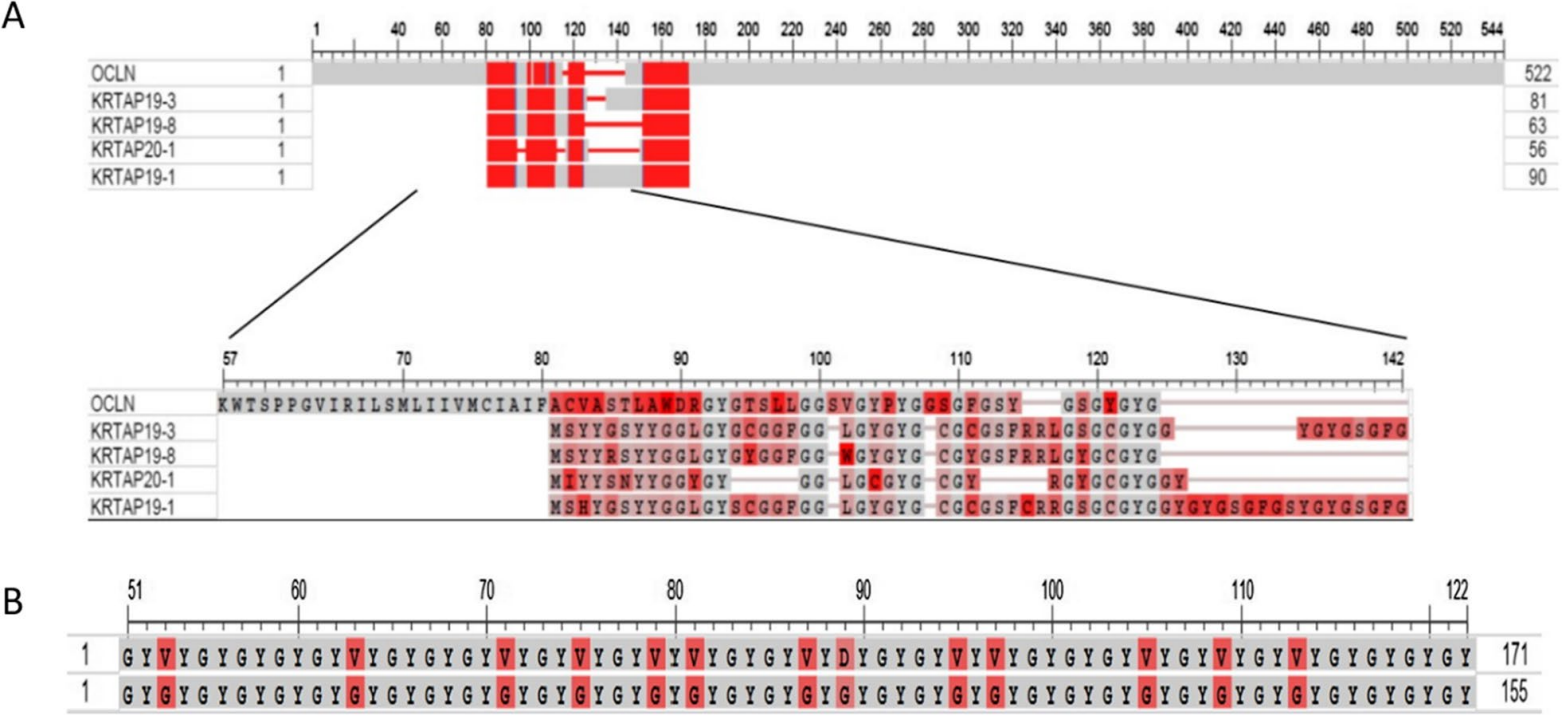
**A** Alignment plot comparing occludin and four occludin-linked KRTAP proteins of *Homo sapiens*. Upper portion—full sequence. Lower portion—a higher matched section on an expanded scale. (The colour coding here is the “Column Quality score–Protein”, which assigns scores to residues based on how well a particular residue agrees with the others in the column (grey high to red low). The KRTAPs are, from top downwards: 19–3, 19–8, 20–1, and 19–1. The Expect values of the four KRTAPs against occludin are 2 × 10^–05^, 3 × 10^–05^, 4  × 10^-05^, 7  × 10^-05^, and 5  × 10^-04^, respectively. The occludin itself lies at the top of the set (Q16625).  **B **Alignment plot comparing the sequence of the KRTAP21-1-like protein of the bank vole *Myodes glareolus* (upper row) with the GY repeating sequence that is the KRTAP6-2-like protein of the collared flycatcher (lower row)

Figure 4 below shows the alignment plot for occludin and some occludin-linked KRTAPs:

4 × 10^–05^, 7 × 10^–05^, and 5 × 10^–04^, respectively. The occludin itself lies at the top of the set (Q16625). **B** Alignment plot comparing the sequence of the KRTAP21-1-like protein of the bank vole *Myodes glareolus* (upper row) with the GY repeating sequence that is the KRTAP6-2-like protein of the collared flycatcher (lower row).

Note in Fig. 4A, top portion, that the whole of the sequences of these KRTAPs are found to be embedded within the sequence of the occludin protein. Although it is only a small portion of the occludin protein that is matched by these sequences in the KRTAP, this is sufficient to suggest a sequence similarity between these KRTAPs and occludin. Note that many of the glycine (G) and tyrosine (Y) residues in the occludin that align with those in the KRTAP are found in the triplet GYG or in the quintuplet GYGYG. Indeed, the presence of the GYG triplet is practically a defining feature of the O lineage. All except KRTAP7-1 of the O lineage have one-seventh or more of their amino acids in a GYG triplet.

### Properties of the O and M lineage

KRTAPs *compared.* The O and M lineages differ in a number of their characteristics. Properties of the 93 KRTAPs are listed in Additional file 3: Tsble S1.

Additionally, as listed in Additional file 3: Tsble S1, headed “InterPro Domain”, all of the proteins that possessed the “Uniprot IPR002494﻿, KAP” domain were of the M lineage, whereas none of those of the O lineage did so. Five members of the M lineage were not listed with this domain: KRTAPs 3–1,3–2, and 3–3 and also 17–1 and 12–2. All except one of the members of the O lineage were listed with the IPR021743, KRTAP_type6/8/16/19/20/21 domain. The deviant protein here was 7–1, which was noted above as having a lower than typical content of the GYG triplet. The lengths of the proteins of the two lineages are different. The M lineage has a median length of 185 residues (156 to 251, 25% to 75%), while that of the O lineage is smaller at a median of 71.5 (63 to 84, 25% to 75%), significantly different at *P* < 0.001, (Mann–Whitney Rank Sum Test). The two lineages differ also in chromosome location (see Additional file 3: Tsble S1, columns headed “Chromosome” and “Transcript start (bp)”). The O lineage resides on a contiguous section of chromosome 2; the M lineage resides on chromosome 11 as well as on 21.

Although the members of the KRTAP5 family are in the M lineage by many of the criteria that we have described above, they do differ from the remaining M lineage proteins in a number of ways, and might be considered as a sub-lineage of the Ms, even perhaps as the 5 lineage. For instance, they are located apart from the other Ms, all the 5 s being on chromosome 11. They are listed as ultrahigh sulphur (as are some other members of the M lineage) but some 10% of that sulphur is in the sequence CCCKPVCC (or in one case, CCCKPMCC), a sequence not found in any other KRTAP. When, in the following section of this manuscript, the tissue distribution of the KRTAPs is described, it will be noted that the KRTAP 5 family has a characteristic pattern, unlike that of other members of the M lineage or of the O lineage.

### The influence of lineage on the tissue expression of the KRTAPs in humans

Expression data are listed in Additional file 3: Tsble S1, column “Total Skin expression”, culled from a study by Tsoi et al. [19] (see "Methods" section). We extracted from these data the M lineage and O lineage cases and analysed, by an Anova on ranks, the Total skin expression data as a function of lineage. The result was as Median, 25% to 75%: for the M lineage 189.1, 88.56 to 277.36; for the O lineage 7.29, 2.53 to 72.16, significantly different at *P* < 0.00 1.(The difference, by the same statistical test, between the expression values for the members of the *KRTAP* family 5, and the values for the M lineage with the family 5 members removed, was not significant at *P* = 0.764).

Additional file 3: Tsble S1, column “Uniprot: Tissue specificity” lists for each *KRTAP* gene the tissue specificity, culled from the Uniprot database (see "Methods" section). Searching with “hair” gave (only) 52 hits of which 40 were classified under Column “Source” as M direct and 4 were O direct, the difference being significant, by the z test, at *P* = 0.020. The phrase “restricted to hair root” returned 8 hits, all except 1 being from the M lineage. Of these 7, 4 were from the KRTAP5 family. The phrase “Expressed in the hair follicles” Is exclusively associated with the M lineage and specifically with the *KRTAP4*s of this lineage. All of the *KRTAP4*s except two are associated with this tissue specificity grouping.

The category “Restricted to a narrow region of the hair fiber cuticle, lying approximately 20 cell layers above the apex of the dermal papilla of the hair root; not detected in any other tissues” Is largely a property of the *KRTAP10* family of the M lineage. Of the 12 genes with this designation, 8 are *KRTAP10*s while 4 are *KRTAP12*s (these being all the four members of the KRTAP12 family, whose source lies in both the metallothionein-indirect and occludin-indirect groups).

Finally, on separating out those proteins that were listed as “also expressed in other tissues”, 31 were designated as from the O or M lineages. Of these, 19 were from the 53 genes of the M lineage, 12 were from the 16 Os. As a fraction of their occurrence, that of the O lineage genes was significantly higher, by the Z test, at *P* = 0.035.

Thus the O lineage genes and the *KRTAP5* family of M lineage genes were at opposite poles of the tissue specificity spectrum, the O lineage being, in particular, found as expressed in tissues other than hair, while the eleven *KRTAP5* family genes were very narrowly expressed, four being “Restricted to hair root” while seven were listed as “Restricted to a narrow region of the hair fiber cuticle, lying approximately 20 cell layers above the apex of the dermal papilla of the hair root; not detected in any other tissues”.

We analysed in similar fashion a study with some clinical significance that compared *KRTAP* expression on the scalps of patients with alopecia with those of healthy subjects [20]. In both sets of data, the expression of genes of the M lineage was some 50% higher than those of the O lineage genes, statistically so (Mann–Whitney Rank Sum Test test, *P* < 0.001), in both cases. It was, however, the expression of the M lineage genes that differed between the Alopecia and healthy subjects: Median (25% to 75%); Healthy 14.09 (12.8 to 15.2); Alopecia 11.88 (10.8 to 13.1), difference significant at *P* < 0.001. The corresponding difference for the O lineage genes was non-significant at *P* = 0.83.

#### Evolutionary history of the occludin-linked and metallothionein-linked KRTAP proteins

**Fig. 5:**
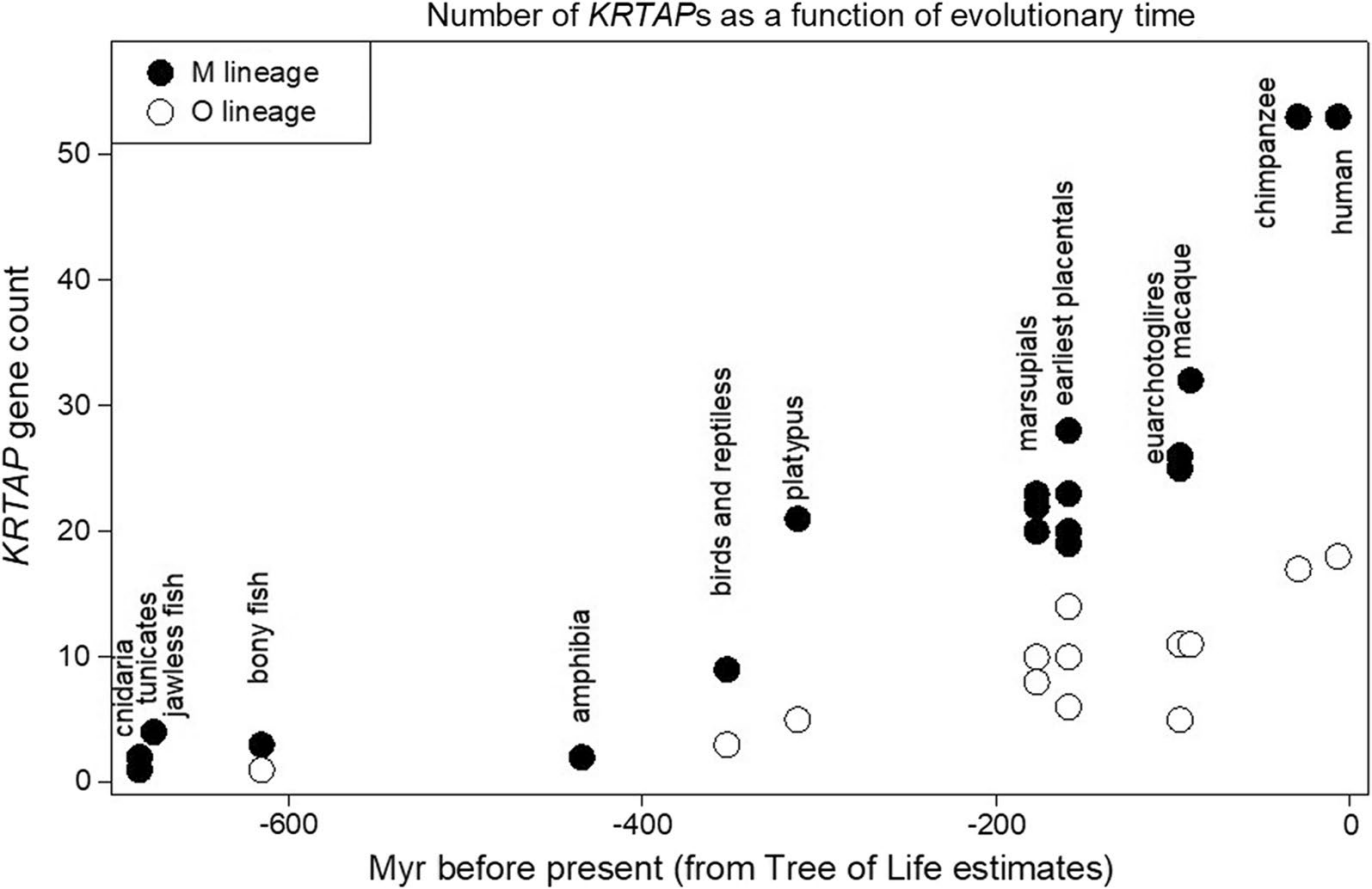
The evolutionary history of the occludin- and metallothionein-linked KRTAPs. Y-axis: the number of KRTAPs found by a BLAST search of the denoted animals, using as bait either occludin—O lineage (open circles) or a metallothionein—M lineage (filled circles). The time when the denoted clade or animal species first appeared is given on the X axis

Our BLAST searches against the human genome had revealed 16 of these proteins to be closely related to occludin and 53 to be closely related to metallothionein, and we defined these two groups as the members of the O lineage or the M lineage, respectively. We extended these observations to embrace a series of animal genomes ranging from the sea anemone of phylostratum 6 through to the human and chimpanzee of phylostratum 19, the primates. The results of these searches are collected in the Additional file 1. Figure 5 depicts the results of these studies from the Cnidaria through to the humans.

It would appear that the number of O lineage KRTAPs, those that are linked by sequence similarity to occludin, increases steadily from the amniota (reptiles and birds) through to the great apes, and is always lower than the number of those of the M lineage (linked by similarity to metallothionein).

**Fig. 6:**
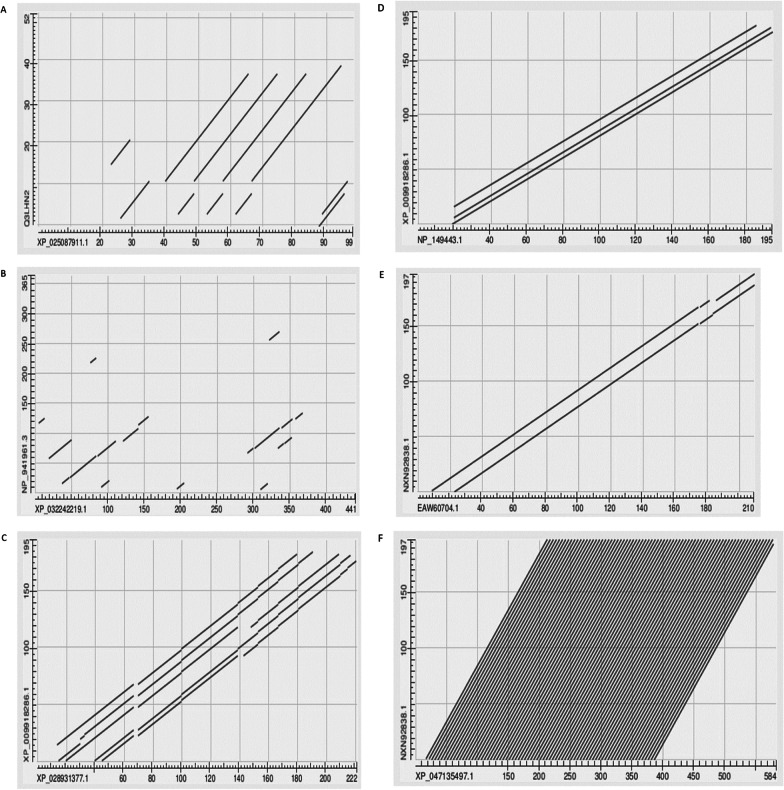
Dot plots derived from BLAST 2-sequences comparisons. **A** the relation between, on the x-axis, XP_025087911.1, the KRTAP19-2-like protein of the golden apple snail *Pomacea canaliculata,* and, on the Y-axis, Q3LHN2, the KRTAP19-2 protein of *Homo sapiens;* **B** on the x-axis, XP_032242219.1, the KRTAP5-1-like protein of the Sea anemone, *Nematostella vectensis,* and, on the Y-axis, NP_941961.3, the KRTAP10-6 protein of *Homo sapiens*; **C** on the x-axis, XP_028931377.1, the platypus KRTAP4-3-like isoform X1*,* and, on the Y-axis, XP_009918286.1, KRTAP4-3 from the white-tailed sea eagle, *Haliaeetus albicilli*; **D**: on the x-axis, NP_149443.1, the KRTAP4-3 of *Homo sapiens,* and, on the Y-axis, XP_009918286.1, KRTAP4-3 from the white-tailed sea eagle, *Haliaeetus albicilli*; **E**: on the x-axis, EAW60704.1, the KRTAP4-7 of *Homo sapiens,* and, on the Y-axis, NXN92838.1, the protein KRA43 of the scimitar bird, *Rhinopomastus cyanomelas*; **F**: on the x-axis, XP_047135497.1, the KRTAP10-4-LIKE of *Hydra vulgaris,* and, on the Y-axis, NXN92838.1, the protein KRA43 of the scimitar bird, *Rhinopomastus cyanomelas.* NOTE: Alignments between the two sequences in each dot plot of Fig. 6 A through F are collected as a Powerpoint labelled Additional file 2.

Examples of top hits found when searching non-mammalian species for the early presence of KRTAP homologues of members of the O and M lineages are listed in Table 1, together with the appropriate pair-wise BLAST 2-sequence Expect values. Dot plots of some of the pairs are depicted in Fig. 6. The KRTAP6-2-like protein of the collared flycatcher *Ficedula albicollis* in that list has a most unusual sequence. Of its 155 amino-acid residues, 123 form a continuous chain of the pair glycine.tyrosine (G.Y). In Fig. 4B earlier, the upper row depicts this GY repeating sequence that is the KRTAP6-2-like protein of the collared flycatcher while the lower sequence in that figure shows the sequence of the KRTAP21-1-like protein of the bank vole Myodes glareolus,. An accurate Expect value could not be computed because of the very high degree of low complexity sequence. Following the suggestion of Jarnot et al. [21, 22] we performed the BLAST search with “No adjustments” and the low complexity filter unchecked. This gave us an Expect value which we do not quote since it should not be compared with the other Expect Values reported in this paper, but we were able to retrieve a value for the percent identity between the two sequences: 83.33%.

**Table 1:** Comparing mammalian KRTAPs with their homologues in other species (examples from Fig. 5)

Mammalian keratin-associated protein	Homologue in non-mammalian species	Expect value in BLAST 2-sequence comparison
Human^1^ KRTAP10-6	Sea anemone^9^ KRTAP5-1-like	3 × 10–03
Human^1^ KRTAP19-2	Snail^7^ KRTAP19-2	1 × 10–05
Human^1^ KRTAP19-2	Scallop^8^ KRTAP6-2-like	2 × 10–04
Human^1^ KRTAP2-1	Chicken^11^ KRTAP5-5-like	2 × 10–05
Human^1^ KRTAP22-2	Wren^4^ KRTAP21-1	3 × 10–05
Human^1^ KRTAP22-2	Flycatcher^5^ KRTAP6-2-like	6 × 10–05
Human^1^ KRTAP4-3	Scimitar bird^12^ KA43	8 × 10–05
Human^1^ KRTAP4-7	Sea eagle^13^ KRTAP4-3	8 × 10–05
Human^1^ KRTAP6-2	Salmon^3^ KRTAP6-2	8 × 10–05
Human^1^ KRTAP6-3	Lizard^6^ KRTAP6-2	3 × 10–04
Platypus^14^ KRTAP4-3	Sea eagle^13^ KRTAP4-3	1 × 10–32
Vole^2^ KRTAP21-1	Puffer fish^15^ KRTAP21-1	1 × 10–100
Vole^2^ KRTAP9-1	Starfish^10^ KRTAP5-1-like	6 × 10–04

^1^*Homo sapiens* ^2^*Arvicola amphibious* ^3^*Oncorhynchus keta* ^4^*Acanthisitta chloris* ^5^*Ficedula albicollis* ^6^*Anolis carolinensis* ^7^*Pomacea canaliculata* ^8^*Pecten maximus* ^9^*Nematostella vectensis* ^10^*Asterias rubens* ^11^*Gallus gallus* ^12^Rhinopomastus cyanomelas ^13^ Haliaeetus albicilli ^14^*Ornithorhynchus anatinus* ^15^*Takifugu rubripesI*

In contrast, in the M lineage, a metallothionein-linked protein, KRTAP 10-1-like, is found already in the sea anemone *Nematostella vectensis* (where a KRTAP5-1-like protein with an Expect value of 0.003, when matched against the KRTAP10-6 of *Homo sapiens* was found in a BLAST 2-sequences determination—see Fig. 6B). Also in the starfish, *Asterias rubens*, an M lineage protein, KRTAP4-7-like, matched against KRTAP9-1-like of the European water vole, *Arvicola amphibius* gave an Expect value of 6 × 10^–04^ in a BLAST 2-sequences determination.

The evolutionary history of the KRTAP5 family of the M lineage is of particular interest. While proteins annotated as KRTAP5-like can be found already in the starfish, the sequence CCCKPVCC that we previously described as characteristic of the KRTAP5 family members of *H. Sapiens,* is still absent in the platypus of phylostratum 15 but first appears in the marsupials, late in that phylostratum.

Included on Fig. 5 are the metallothionein-linked proteins, annotated as KRTAPs, that we found in the chicken. One of these proteins, assigned the name KRTAP5-5-like, had an Expect value in a BLAST 2-sequences determination against the KRTAP2-1 of *Homo sapiens* of 2 × 10^–05^. Another bird-derived protein, KRTAP4-3 from the white-tailed sea eagle, *Haliaeetus albicilli* was found to have an Expect value of 1 × 10^–32^ when compared against the platypus KRTAP4-3-like isoform X1 (Fig. 6C), and of 2 × 10^–49^ against the KRTAP4-3 of *Homo sapiens* (Fig. 6D). The protein annotated as KRA43 of the scimitar bird, *Rhinopomastus cyanomelas,* returned an Expect value of 5 × 10^–48^ against the KRTAP4-7 of *Homo sapiens* (Fig. 6E)*,* all of these being in BLAST 2-sequences determinations.

When the scimitar bird’s KRA43 sequence was used as the bait in a BLAST search of the human genome, 19 of the top 20 hits in the list returned were to members of the 22-member strong KRTAP4 family (the single remaining hit was to the unannotated sequence EAW6070). The KRTAP4 family members possess multiple repeats of various sequences, each such sequence beginning with CC followed by a dozen or so other amino acids (see Additional file 3). The sequence from the scimitar bird was itself highly repetitive being largely composed of repeats of the sequences CCRPT and QTDLL. Further down in the list of BLAST hits were members of the KRTAP9 and KRTAP10 families. Comparing human KRTAP9-1 and the scimitar protein returned an Expect value of 6 × 10^–30^, while against human KRTAP 10–9, the Expect value was 9 × 10^–31^. The KRTAP9 and KRTAP10 families, too, possess multiple copies of various repeated sequences, always beginning with CC. Interestingly, nestling among these latter hits was a keratin, sequence CAA44938.1, annotated as “high sulphur keratin”. A BLAST 2-sequences comparison of this keratin with the sequence from the scimitar bird returned an Expect value of 3 × 10^–18^ suggesting a strong similarity between this sequence of a keratin and the sequence of a KRTAP. Among the lower animals, the KRTAP10-4-like protein of the Cnidarian *Hydra vulgaris* demonstrated an Expect value of 4 × 10^–60^ in a BLAST 2-sequence comparison with the sequence from the scimitar bird. The protein from Hydra is almost entirely composed of repeating sequences of CCTDY. A dot plot of the relation between these two sequences is given as Fig. 6F above.

These data hint at a common origin for the KRTAP4, KRTAP9 and KRTAP10 families, reaching back at least to the Cnidaria.

Repeated sequences of low complexity are a common feature of the KRTAPs. Column J of the Table found in Additional file 3: Table S1 provides a list of the repeated sequences found in the KRTAPs. 72 of these proteins possess multiple copies of repeated low complexity sequences, the highest number being found in KRTAP 10–4 which has 36 repeats of five-membered sequences CC***. (Rado-Trilla and Alba [22] have pointed out the importance of low-complexity sequences in the evolution of vertebrate proteins. The expansion of protein families is frequently associated with increases in the number of low complexity sequences). An extreme example of a KRTAP possessing multiple low complexity repeats, that of the KRTAP5-6-like-protein of the collared flycatcher with its 123 continuous sequence of the GY pair was discussed above.

### Expression of KRTAP proteins in non-mammalian organisms

**Fig. 7:**
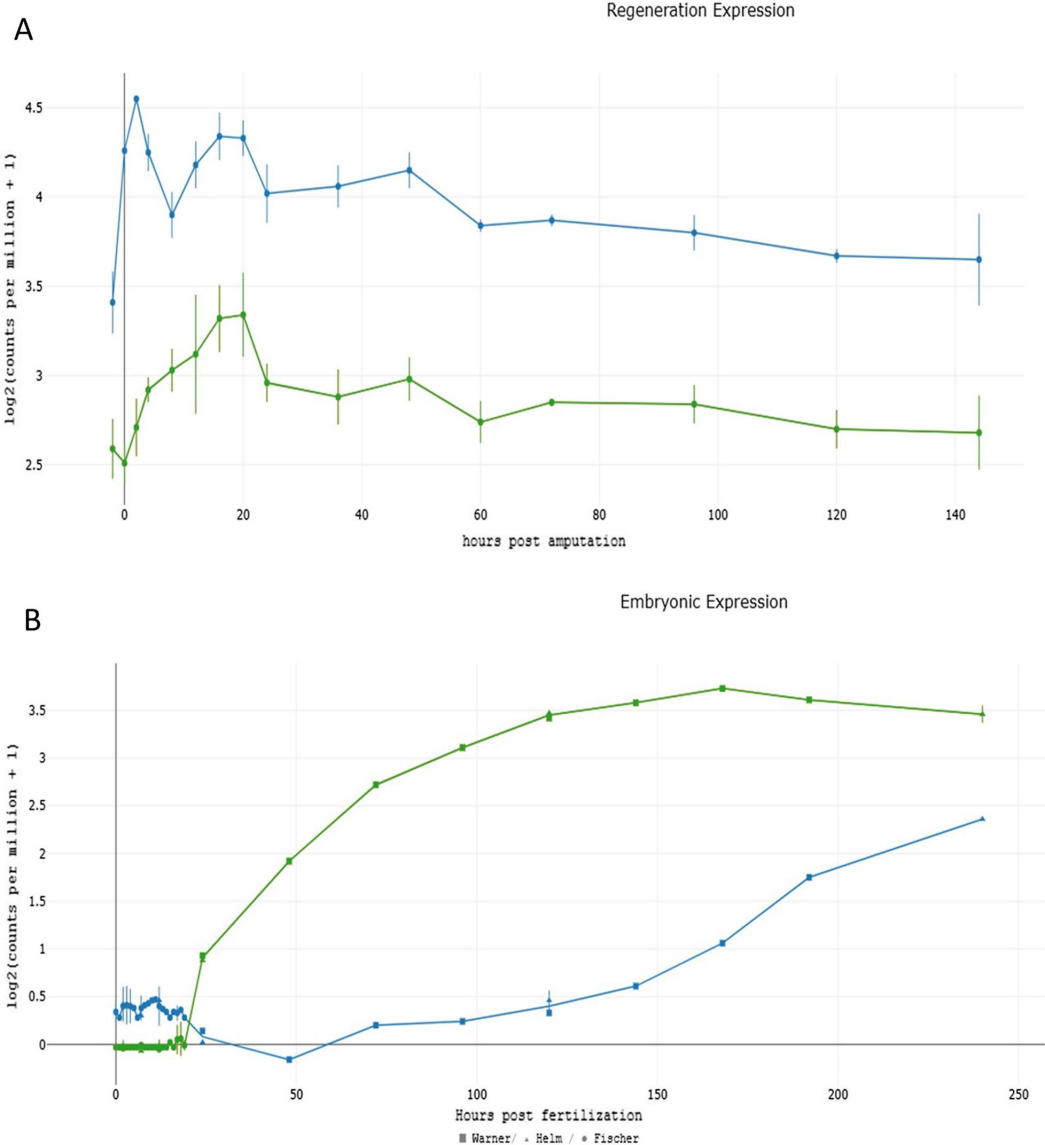
Expression of two Nematostella KRTAP-like genes, *NvERTx.4.10414* (blue) and *NvERTx.4.57316* (green) during **A** Regeneration, and **B** embryonic development. The expression values are retrieved from NvERTx, An embryogenesis & regeneration gene expression plotter (http://nvertx.ircan.org/ER/ER_plotter/home).13

Gene expression databases contain numerous examples of the expression of *KRTAP*s in non-mammalian organisms. When we searched for *KRTAP* expression in the sea anemone Nematostella, we found a sequence, *NvERTx.4.10414*, that was identified in the database as closely related to the cow Bos taurus *KRTAP9-1*. A BLAST 2-sequences comparison between the Nematostella sequence and the *KRTAP9-1* of Bos taurus returned an Expect value of 1 × 10^–08^. Figure 7A depicts the gene expression pattern of *NvERTx.4.10414* as a function of time during the regeneration of the sea anemone, and of a second *KRTAP10-6*-like related protein of the sea anemone, *NvERTx.4.57316* during the same process. The embryonic expression of the two Nematostella *KRTAP* like genes is shown in Fig. 7B and reveals time-dependent (post fertilization) differences in their expression levels.

It should be noted that the gene *KRTAP9-1* is expressed at a medium level in the hair follicle and skin of the mouse (Mouse Genome Informatics MGI-Mouse Gene Expression Database (GXD) (jax.org). Interestingly, the expression of *KRTAP10-6* is given in Additional file 3: Table S1 as “Restricted to a narrow region of the hair fiber cuticle, lying approximately 20 cell layers above the apex of the dermal papilla of the hair root; not detected in any other tissues”.

**Fig. 8:**
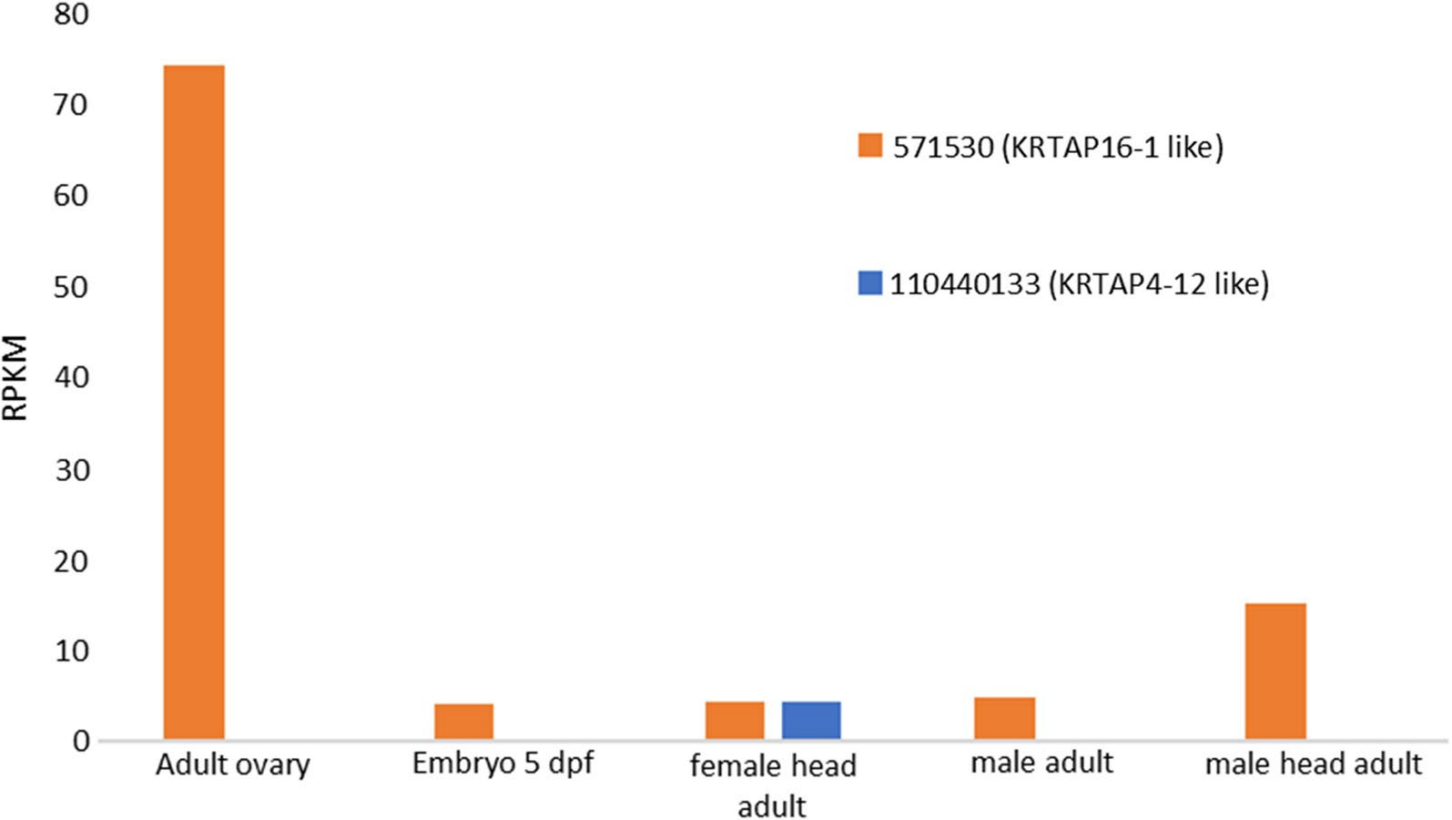
Expression of two Danio rerio *KRTAP*-like genes, *571530* (orange) and *110440133* (blue) in different tissues and developmental stages of the zebrafish. The expression values were retrieved from NCBI: https://www.ncbi.nlm.nih.gov/gene/110440133/?report=expression and https://www.ncbi.nlm.nih.gov/gene/571530/?report=expression)

Expression of KRTAP-like proteins is also reported in the zebrafish *Danio rerio.* Figure 8 shows two such records. One is for a *KRTAP4-12*-like protein (XP_021335863.1, Gene_ID: 110440133), which is expressed in the adult female head. The second is for a *KRTAP16-1*-like protein (XP_700224.3, Gene_ID: 571530) which has highest expression in the ovary. Using the latter zebrafish sequence as bait in a search of the mammals gave as the top hit the KRTAP 10–11 of the Eurasian badger *Meles meles.* A BLAST 2-sequences comparison between this sequence and that of the fish returned an Expect value of 1 × 10^–22^.

Of the two genes shown above to be expressed in *Dani*o, *KRTAP4-12* is expressed in human hair follicles (see Additional file 3: Table S1 and also Marquardt et al. 2015) [23], while *KRTAP16-1* is expressed in human skin and hair (Tissue expression of KRTAP16-1—Summary—The Human Protein Atlas), and is noted also as being expressed in the wool of Hetian sheep [24].

We also found five genes annotated as KRTAP genes in the chicken. As described in the "Methods" section, expression data for Gallus gallus were downloaded from bgee.org, and included the following data sets: GSE30352, GSE41338, GSE41637, SRP057141, SRP092128, and SRP097223. A gene was flagged as “expressed” if it had at least 2 read counts in an RNAseq experiment. Table 2 records the results of this search, the five genes being listed with the organ in which they are found in the bird (four in the kidney and one in colon), the Expect value in a Blast 2 sequences determination of the top hit in a BLAST search of the gene against *H. sapiens* and also the Blast 2 sequences determination against the orthologous gene in *H. sapiens*.

**Table 2:** Expect values from blasts using chicken-expressed krtap sequences

Results of BLAST searches with KRTAP protein*s* of *G. gallus* run against *H Sapiens* genome						
Chicken gene providing expressed sequence	KRTAP sequences in top 1000 hits	Top BLAST hit in *H sapiens*	Its Expect value in a BLAST 2- sequences analysis	Uniprot ID of orthologous protein in *H sapiens*	Its Expect value in a BLAST 2- sequences analysis	Expressed in
*KRTAP10-4*	98	KRTAP5-7	0.054	P60372	0.031	Kidney
*KRTAP 5–1-like*	87	KRTAP9-1	0.038	Q6L8H4	0.31	Kidney
*KRTAP 9–1-like*	86	KRTAP 9–1	0.30	A8MXZ3	0.32	Colon
*KRTAP21-1-like*	17	KRTAP 6–3	0.019	Q3LI58	12	Kidney
*KRTAP 10–4* (2^nd^ example)	87	KRTAP 9–1	5 × 10^–4^	P60372	13	Kidney

Expression data for Gallus gallus were downloaded from bgee.org, and included the following data sets: GSE30352, GSE41338, GSE41637, SRP057141, SRP092128, and SRP097223. Unique identifiers (ENSGALGx) for genes encoding KRTAP-like proteins were found by querying the Ensembl genome annotation for Gallus gallus (Genome version GRCg7b). A gene was flagged as “expressed” if it had at least 2 read counts in an RNAseq experiment

### The early antecedents of keratin-associated proteins that have been shown to be localized in hair- or wool-producing cells

There are a number of cases where keratin-associated proteins have been clearly shown to be localised in the trichocytes that produce hair or wool. We asked whether any of these proteins had antecedents in animals that had evolved before the appearance of hair or wool. Our findings for some of these case are summarized in Table 3, where we list the mammalian protein together with its non-mammalian homologue and the Expect Value for a BLAST 2-sequence comparison between the members of the pair.

**Table 3:** Comparing some mammalian KRTAPs with their non-mammalian homologues

Mammalian keratin-associated protein	Homologue in non-mammalian species	Expect value in BLAST 2-sequence comparison
Sheep^1^ KRTAP 11–1	Darter^3^ KRTAP 4–3-like	2 × 10^–09^
Sheep^1^ KRTAP 3–3	Toucan^4^ KRA43	1 × 10^–04^
Human^2^ KRTAP 3–3	Toucan^4^ KRA43	7 × 10^–04^
Human^2^ KRTAP 5–2	Sorghum^5^ KRTAP 5–2	5.5
Human^2^ KRTAP 19–2	Water lily^6^ KRTAP 19–2	0.4

^1^*Ovis aries* ^2^*Homo sapiens* ^*3*^*Etheostoma spectabile* ^4^*Ramphastos sulfuratus* ^5^*Sorghum bicolor* ^6^*Nymphaea colorata*

#### KRTAP-annotated proteins in plants

We also searched for KRTAP-annotated proteins in plant material. Searching with either the metallothionein MT1A or with occludin, we obtained a number of KRTAP-annotated hits, the two top hits being listed in Table 3. The Expect values indicate a low sequence similarity. The plant proteins contained numerous GYG sequences, and a few GYGYG sequences, such as we had identified previously as being characteristic of the O lineage KRTAPs.

### Early appearance of the ectodermal dysplasia (EDA) signaling pathway

For our discussion on the evolutionary origin of the hair follicle, we needed to determine the earliest appearance of the genes of the ectodermal dysplasia (EDA) signaling pathway. Using again BLAST searching of the genomes of the early animals with the three genes of this pathway, *EDA, EDAR*, and *EDARADD*, we found annotated orthologs of all three in the lamprey *Petromyzon marinus.* These had Expect values in BLAST 2-sequences searches against the lamprey of 2 × 10^–23^, 7 × 10^–21^ and 1 × 10^–29^ for EDA, EDAR, and EDARADD proteins respectively. The tunicate *Ciona intestinalis* lacked the *EDARADD* gene. In the lancelet *Branchiostoma belcheri* the only annotated representative of the three genes was “PREDICTED: ectodysplasin-A-like [*Branchiostoma belcheri*].

## Discussion

### The ancient roots of the keratin-associated proteins

The keratin-associated proteins were first identified during research into the properties of wool in research aimed at the development of this important commercial product. Later they were found also in hair. Both hair and wool are features of the mammals. With the accumulation of knowledge of genomic sequences, genes have been annotated as keratin-associated proteins or KRTAP-like proteins in many non-mammalian organisms. The previous section of this paper has noted some of these, found in the genomes of animals that pre-date by hundreds of millions of years the first appearance of hair or wool in the evolutionary record. Proteins with close similarity to the mammalian KRTAPs (as determined by the low Expect values obtained in BLAST 2-sequences against protein comparisons) are found in fish, in reptiles, in birds and even in molluscs. What can be the role of these allegedly hair or wool proteins in the non-hairy organisms?

On the one hand, that a mammalian KRTAP does bind to a keratin has been clearly demonstrated in physico-chemical studies [2]. They showed, by isothermal titration calorimetry, that KRTAP8-1, an O lineage protein, can bind directly to the head domain of the trichocyte’s intermediate filament protein, keratin K85. Interactions between other KRTAPs and keratins were also demonstrated. But there is strong evidence that KRTAPs may play a role other than that of being concerned with hair. A genome-wide search for proteins linked to mammary cancer found KRTAP5-5 to be a regulator of cytoskeletal function that modulates cell motility and thus can lead to vascular invasion [26]. Also, numerous KRTAPs were identified among the proliferation drivers in a screen for proliferation regulators in multiple cell types [27]. (All of these KRTAP proliferation drivers were in the M lineage grouping). In addition, as we noted in the "Results" section, a majority of the O lineage KRTAPS were listed in the Uniprot database as “also expressed in other tissues”.

One would not be surprised to find, taking one example, that the “keratin-associated protein–10–1 like” protein in the lancelet *Branchiostoma belcheri*, an animal lacking hair, scales, or quills, might bind to and interact with its keratin. Keratin is expressed in the epithelia of lancelets, beginning in the larval stage [28]. Lancelets also possess multiple intermediate filaments which seem to represent a relatively unspecialized ancestral condition [29]. Thus, KRTAPs were present, and presumably playing a functional role, in organisms that existed long before there is evidence for the evolution of hair. What additional genes were harnessed to enable the evolution of the hair follicle?

### Ectodermal dysplasias and the developmental role of placodes

In his book “The variation of plants and animals under domestication”, Charles Darwin [30] wrote of a communication that he had received from Mr. W. Wedderburn “of a Hindoo family in Scinde in which ten men, in the course of four generations, were furnished in both jaws taken together, with only four small and weak incisor teeth and with eight posterior molars. The men thus affected have little hair on the body and become bald early in life. They also suffer much during hot weather from excessive dryness of the skin. It is remarkable that no instance has occurred of a daughter being thus affected. Though daughters in the above family are never affected, they transmit the tendency to their sons; and no case has occurred of a son transmitting it to his sons.” This was the first published description of what are now known as hypohidrotic ectodermal dysplasia. The ectodermal dysplasias are a group of genetic disorders in which two or more of ectodermally derived structures (the skin, sweat glands, hair, nails, teeth and mucous membranes) develop abnormally. Each person with an ectodermal dysplasia may have a different combination of defects. Thadani [31, 32], working in the Hindu Amil community of Hyderabad Sind with the “Budhas”, the local term for men born toothless, showed that the condition was brought about by an X-linked mutation. The genetic basis of the disease has since been identified as due to mutations in one or other of the three interacting genes *EDA, EDAR* and *EDARADD* [33]. These comprise the ectodysplasin (EDA) pathway which takes part in the embryological development of ectodermal organs including teeth, hairs, feathers, and mammary glands. EDA is a ligand that belongs to the tumor necrosis factor (TNF)-α family. EDAR is its receptor, related to the TNFα receptors. EDARADD is as an adaptor and links EDAR to the downstream NF-κB pathway, regulating the target genes of this pathway.

Ectodermal organs, which include teeth, hair follicles, mammary ducts, and glands such as sweat, mucous and sebaceous glands, are initiated in development as placodes, which are epithelial thickenings that invaginate and bud into the underlying mesenchyme [34]. The signaling pathways involved in placode and bud formation include the Wnt, fibroblast growth factor (Fgf), hedgehog (Hh), transforming growth factor β (Tgfβ), and the bone morphogenetic protein (Bmp) pathways that are key regulators of nearly all organ systems. Working together with them, the tumor necrosis factor (Tnf) family ligand ectodysplasin (EDA) and its serial receptors EDAR and EDARADD have a specific role in ectodermal appendage morphogenesis. Indeed, inactivation of the EDA pathway abrogates primary hair placode formation. [35]

A comprehensive study of the evolution of the EDA pathway [36] found its origin to have been in the jawless fishes, consistent with our BLAST searches as presented in the "Results" section (where annotated low Expect value orthologs of the three genes were found in the lamprey *Petromyzon marinus*). The jawless fish are the first animals to possess teeth, these being located in the pharynx in the lamprey. By the emergence of the jawed fish, teeth had moved to the jaw [34], but in both locations the EDA pathway is utilized in the development of the tooth-forming placodes. The jawless fish do not have scales, scales as a body covering emerging with the jawed fish. Scale development originates in placodes which use the EDA pathway as shown by the fact that loss of the *EDAR* gene leads to the almost complete loss of scales in the Japanese rice fish, Medaka (*Oryzias latipes*) [37]. Reptilian scales also develop from placodes, again involving the EDA pathway [35], with the scale-less form of the bearded dragon (*Pogona vitticeps*) being deficient in the EDA protein. Indeed, Di-Poi and Milinkovich [35] conclude that “most skin appendages in amniotes are homologous; that is, they all evolved from a shared common ancestor that exhibited appendages developing from an anatomical placode and expressing a set of signaling molecules still involved in the development of scales, hairs, and feathers of extant species.”

With the placode-forming process firmly in existence, the therapsids and then the mammals were in the initial stage of their evolution beyond the reptiles. It is not too far-fetched to postulate that the co-option of what we have shown to be ancient lineages of KRTAP-like proteins would allow the emergence of the hair follicle. There, interactions between existing cellular keratins and early versions of the newly co-opted KRTAPs would enable the keratin aggregates, previously lying parallel to the skin surface, now to take up a perpendicular stance, leading to the extrusion of hair, perhaps first in the form of tactile whiskers. As evolution proceeded and new KRTAPs emerged, the varieties of hair and fur types would be developed.

## Supplementary Information

**Additional file 1.** BLAST strategy and results.

**Additional file 2. **Sequences and alignments for dot blots.

**Additional file 3. Table S1. **Characterisation of the keratin-associated proteins.

**Additional file 4: Table S2.** List of the 19 Phylostrata

**Additional file 5: Table S3.** Contenders for the role as ancestral proteins.

## Data Availability

The paper reports no new data, being based on analyses of public databases. The analyses that we made are presented within the paper itself, so no further deposition is necessary. The public databases used were: For KRTAP gene expression, we queried the GEO (Gene Expression Omnibus, https://www.ncbi.nlm.nih.gov/geo/) and retrieved the human skin transcriptomics dataset with accession GSE121212. as well as expression data from Danio rerio (BioProject: PRJEB1986). For Nematostella-specific gene expression data, NvERTx (http://nvertx.kahikai.org) was queried. Tissue specific KRTAP expression information was pulled from Uniprot (www.uniprot.org) and is documented in Additional file 3. All data generated or analysed during this study are either referenced by their accession number or included in this published article and its additional files.
