# Supplementary material for: Ancient lineages of the keratin-associated protein (KRTAP) genes and their co-option in the evolution of the hair follicle
Source: BMC Ecol Evol. 2023 Mar 20;23:7. doi: 10.1186/s12862-023-02107-z (PMC10029157; doi:10.1186/s12862-023-02107-z)
Supplement: Supplementary file 2 — Additional file 2. Sequences and alignments for dot blots. [file 12862_2023_2107_MOESM2_ESM.pptx]

## Slide 1
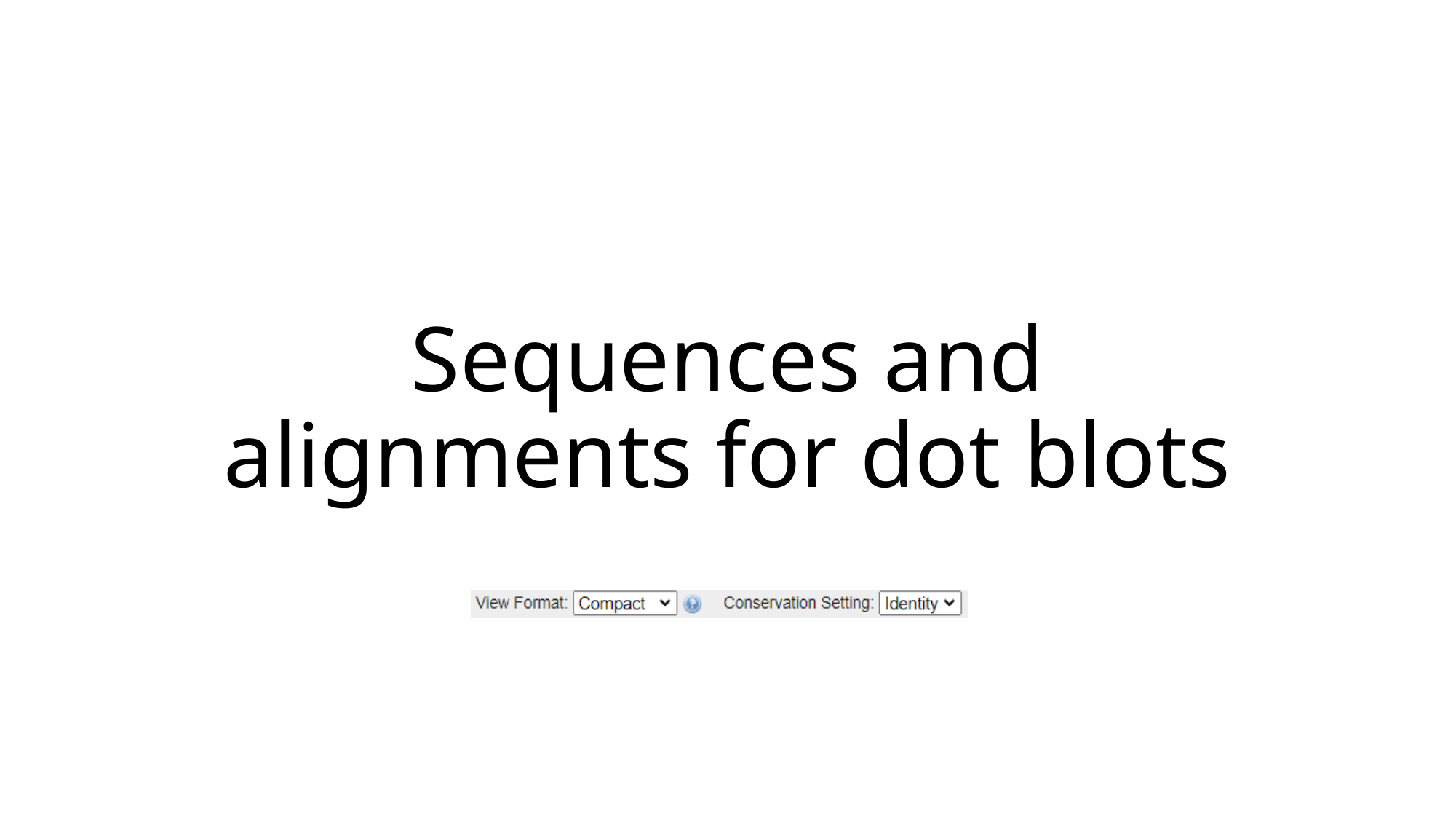

# Sequences and alignments for dot blots

## Slide 2
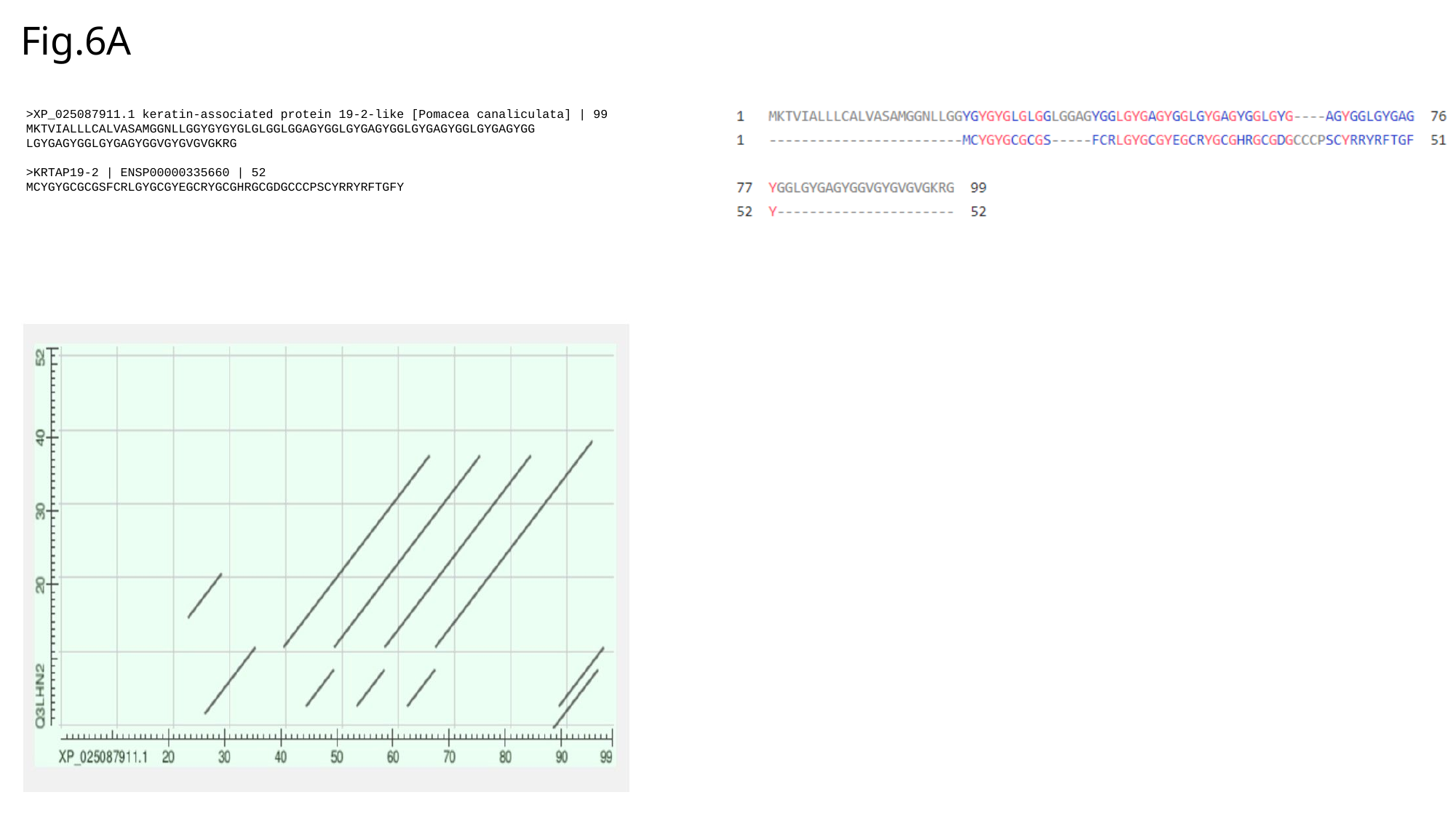

# Fig.6A
>XP_025087911.1 keratin-associated protein 19-2-like [Pomacea canaliculata] | 99
MKTVIALLLCALVASAMGGNLLGGYGYGYGLGLGGLGGAGYGGLGYGAGYGGLGYGAGYGGLGYGAGYGG
LGYGAGYGGLGYGAGYGGVGYGVGVGKRG
>KRTAP19-2 | ENSP00000335660 | 52
MCYGYGCGCGSFCRLGYGCGYEGCRYGCGHRGCGDGCCCPSCYRRYRFTGFY

## Slide 3
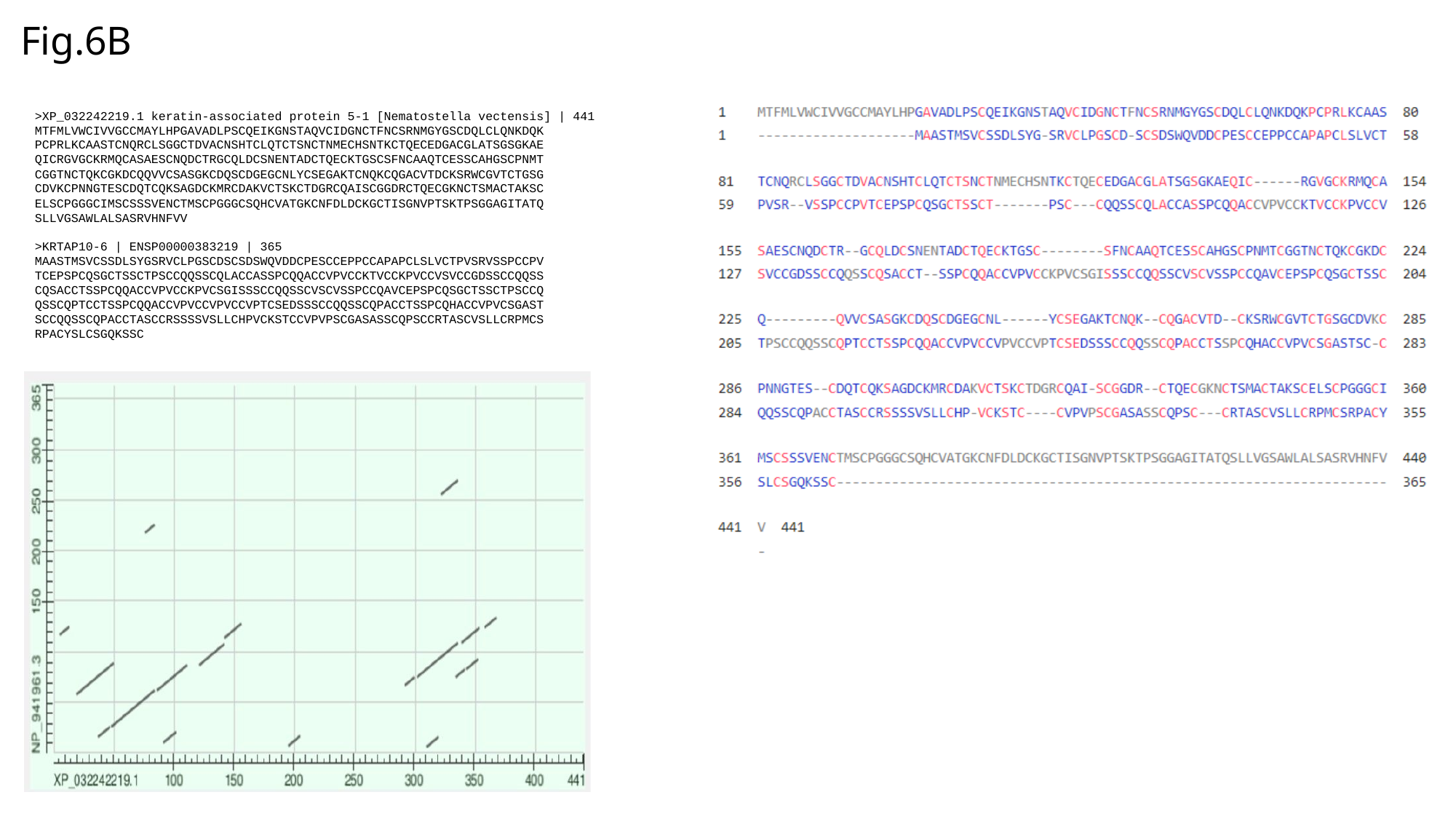

# Fig.6B
>XP_032242219.1 keratin-associated protein 5-1 [Nematostella vectensis] | 441
MTFMLVWCIVVGCCMAYLHPGAVADLPSCQEIKGNSTAQVCIDGNCTFNCSRNMGYGSCDQLCLQNKDQK
PCPRLKCAASTCNQRCLSGGCTDVACNSHTCLQTCTSNCTNMECHSNTKCTQECEDGACGLATSGSGKAE
QICRGVGCKRMQCASAESCNQDCTRGCQLDCSNENTADCTQECKTGSCSFNCAAQTCESSCAHGSCPNMT
CGGTNCTQKCGKDCQQVVCSASGKCDQSCDGEGCNLYCSEGAKTCNQKCQGACVTDCKSRWCGVTCTGSG
CDVKCPNNGTESCDQTCQKSAGDCKMRCDAKVCTSKCTDGRCQAISCGGDRCTQECGKNCTSMACTAKSC
ELSCPGGGCIMSCSSSVENCTMSCPGGGCSQHCVATGKCNFDLDCKGCTISGNVPTSKTPSGGAGITATQ
SLLVGSAWLALSASRVHNFVV
>KRTAP10-6 | ENSP00000383219 | 365
MAASTMSVCSSDLSYGSRVCLPGSCDSCSDSWQVDDCPESCCEPPCCAPAPCLSLVCTPVSRVSSPCCPV
TCEPSPCQSGCTSSCTPSCCQQSSCQLACCASSPCQQACCVPVCCKTVCCKPVCCVSVCCGDSSCCQQSS
CQSACCTSSPCQQACCVPVCCKPVCSGISSSCCQQSSCVSCVSSPCCQAVCEPSPCQSGCTSSCTPSCCQ
QSSCQPTCCTSSPCQQACCVPVCCVPVCCVPTCSEDSSSCCQQSSCQPACCTSSPCQHACCVPVCSGAST
SCCQQSSCQPACCTASCCRSSSSVSLLCHPVCKSTCCVPVPSCGASASSCQPSCCRTASCVSLLCRPMCS
RPACYSLCSGQKSSC

## Slide 4
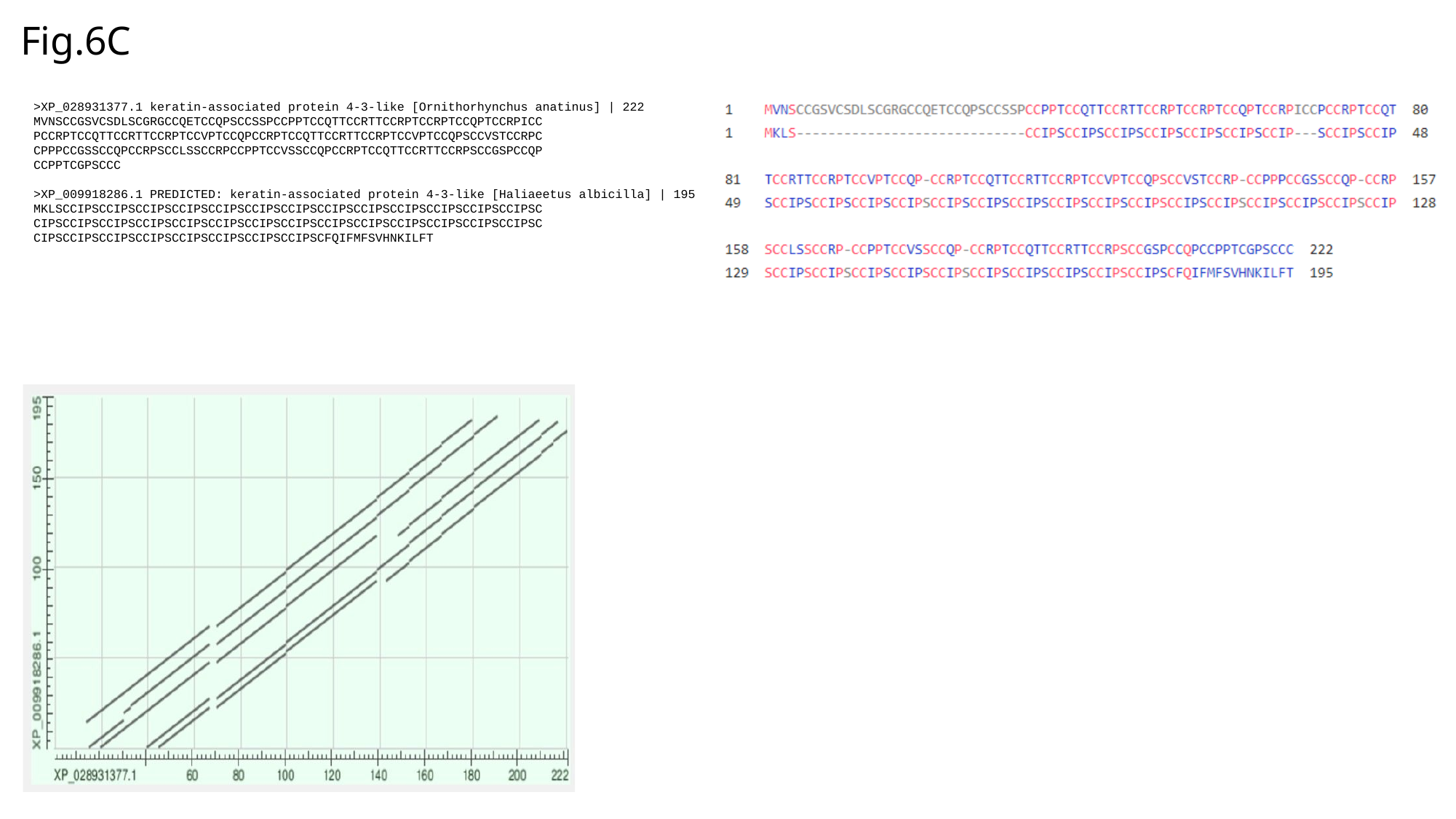

# Fig.6C
>XP_028931377.1 keratin-associated protein 4-3-like [Ornithorhynchus anatinus] | 222
MVNSCCGSVCSDLSCGRGCCQETCCQPSCCSSPCCPPTCCQTTCCRTTCCRPTCCRPTCCQPTCCRPICC
PCCRPTCCQTTCCRTTCCRPTCCVPTCCQPCCRPTCCQTTCCRTTCCRPTCCVPTCCQPSCCVSTCCRPC
CPPPCCGSSCCQPCCRPSCCLSSCCRPCCPPTCCVSSCCQPCCRPTCCQTTCCRTTCCRPSCCGSPCCQP
CCPPTCGPSCCC
>XP_009918286.1 PREDICTED: keratin-associated protein 4-3-like [Haliaeetus albicilla] | 195
MKLSCCIPSCCIPSCCIPSCCIPSCCIPSCCIPSCCIPSCCIPSCCIPSCCIPSCCIPSCCIPSCCIPSC
CIPSCCIPSCCIPSCCIPSCCIPSCCIPSCCIPSCCIPSCCIPSCCIPSCCIPSCCIPSCCIPSCCIPSC
CIPSCCIPSCCIPSCCIPSCCIPSCCIPSCCIPSCCIPSCFQIFMFSVHNKILFT

## Slide 5
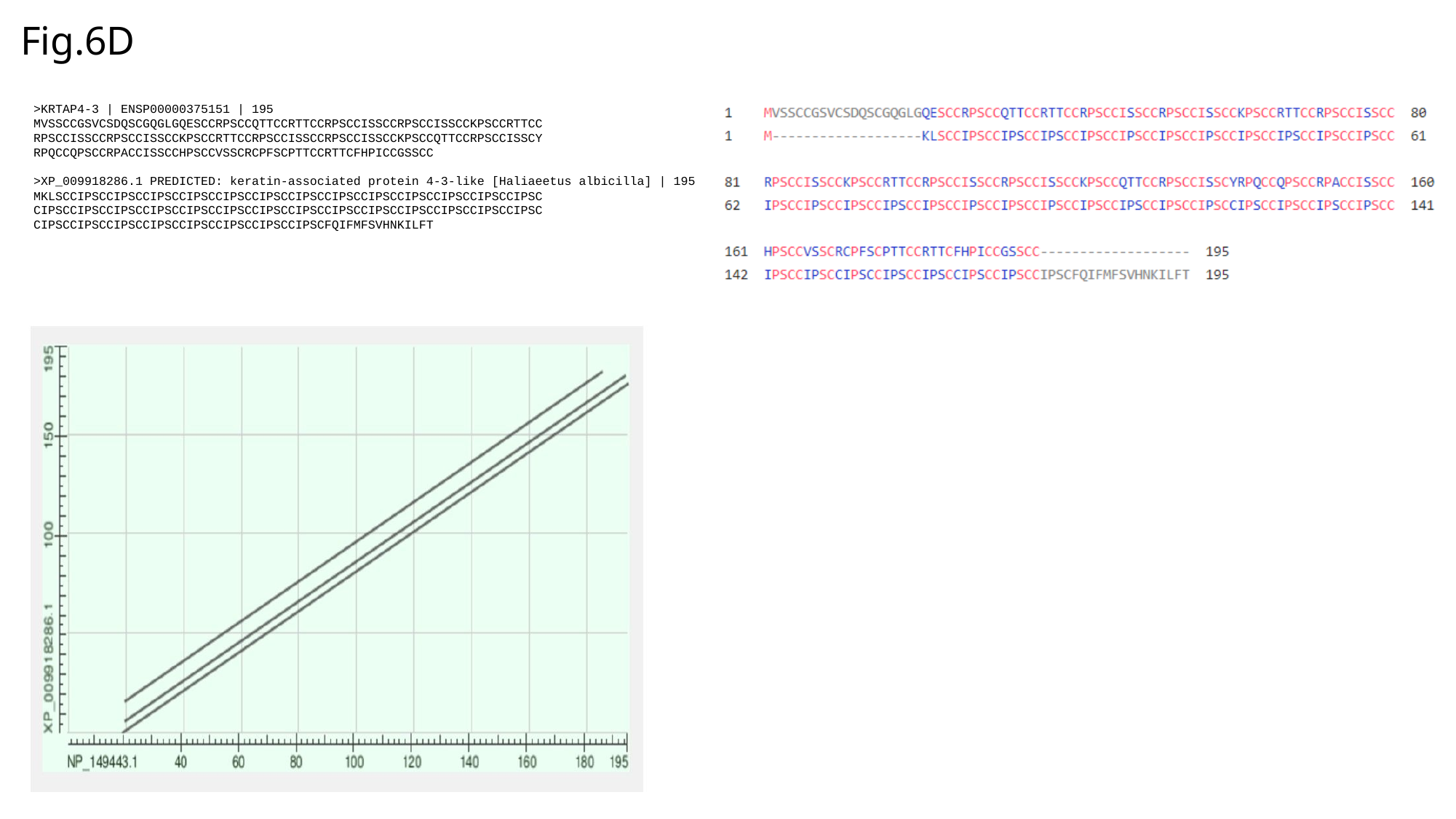

# Fig.6D
>KRTAP4-3 | ENSP00000375151 | 195
MVSSCCGSVCSDQSCGQGLGQESCCRPSCCQTTCCRTTCCRPSCCISSCCRPSCCISSCCKPSCCRTTCC
RPSCCISSCCRPSCCISSCCKPSCCRTTCCRPSCCISSCCRPSCCISSCCKPSCCQTTCCRPSCCISSCY
RPQCCQPSCCRPACCISSCCHPSCCVSSCRCPFSCPTTCCRTTCFHPICCGSSCC
>XP_009918286.1 PREDICTED: keratin-associated protein 4-3-like [Haliaeetus albicilla] | 195
MKLSCCIPSCCIPSCCIPSCCIPSCCIPSCCIPSCCIPSCCIPSCCIPSCCIPSCCIPSCCIPSCCIPSC
CIPSCCIPSCCIPSCCIPSCCIPSCCIPSCCIPSCCIPSCCIPSCCIPSCCIPSCCIPSCCIPSCCIPSC
CIPSCCIPSCCIPSCCIPSCCIPSCCIPSCCIPSCCIPSCFQIFMFSVHNKILFT

## Slide 6
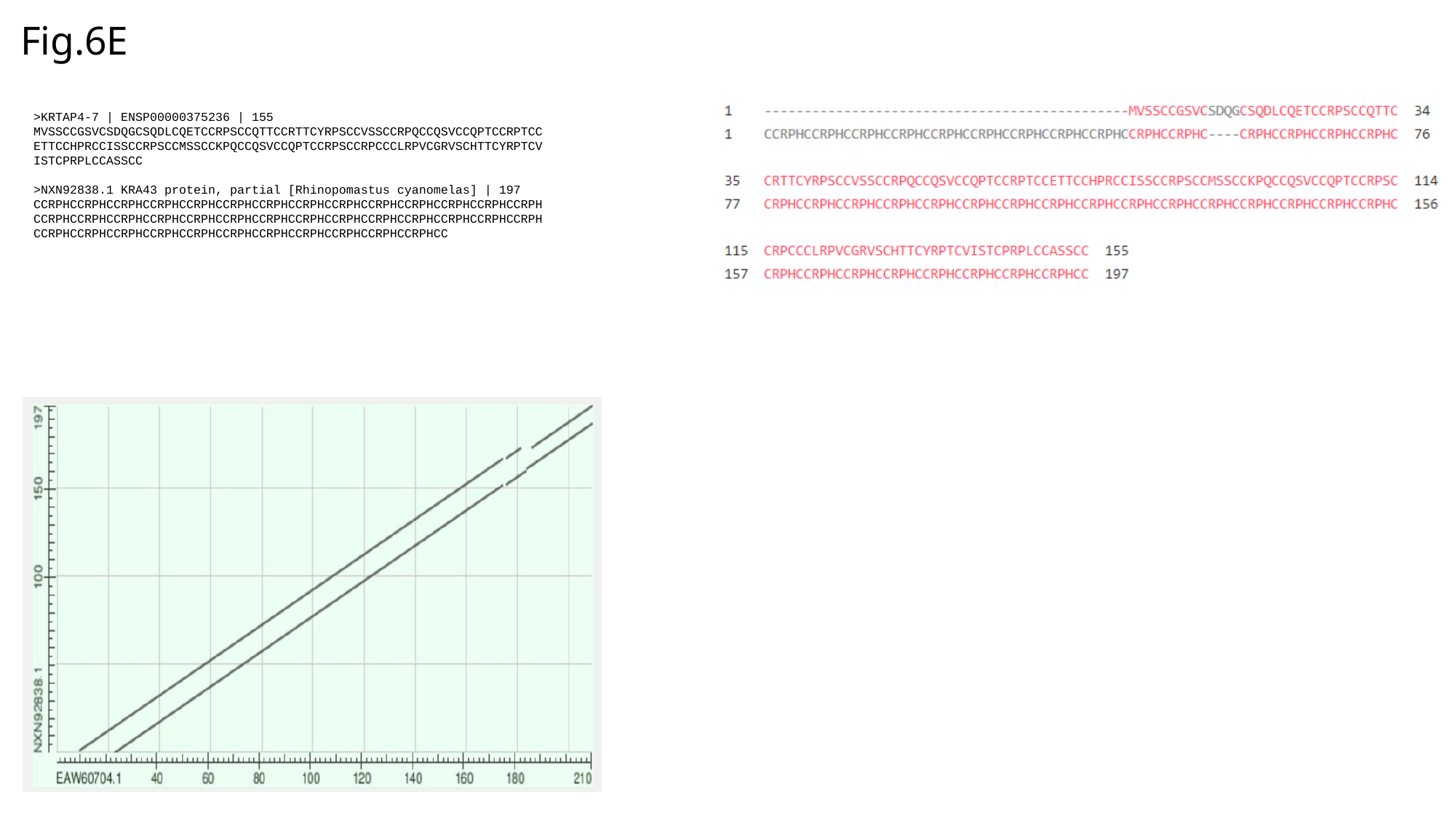

# Fig.6E
>KRTAP4-7 | ENSP00000375236 | 155
MVSSCCGSVCSDQGCSQDLCQETCCRPSCCQTTCCRTTCYRPSCCVSSCCRPQCCQSVCCQPTCCRPTCC
ETTCCHPRCCISSCCRPSCCMSSCCKPQCCQSVCCQPTCCRPSCCRPCCCLRPVCGRVSCHTTCYRPTCV
ISTCPRPLCCASSCC
>NXN92838.1 KRA43 protein, partial [Rhinopomastus cyanomelas] | 197
CCRPHCCRPHCCRPHCCRPHCCRPHCCRPHCCRPHCCRPHCCRPHCCRPHCCRPHCCRPHCCRPHCCRPH
CCRPHCCRPHCCRPHCCRPHCCRPHCCRPHCCRPHCCRPHCCRPHCCRPHCCRPHCCRPHCCRPHCCRPH
CCRPHCCRPHCCRPHCCRPHCCRPHCCRPHCCRPHCCRPHCCRPHCCRPHCCRPHCC

## Slide 7
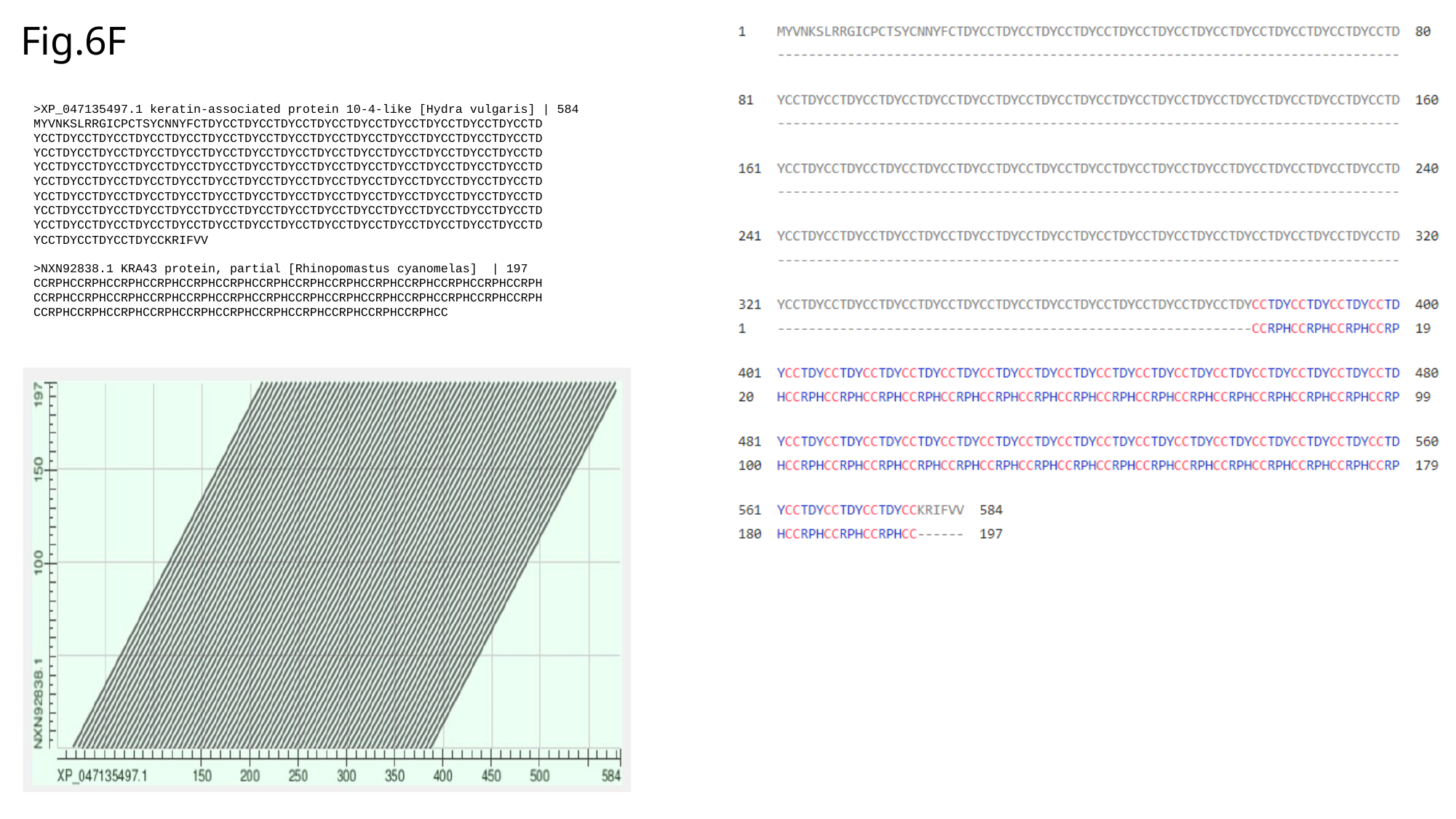

# Fig.6F
>XP_047135497.1 keratin-associated protein 10-4-like [Hydra vulgaris] | 584
MYVNKSLRRGICPCTSYCNNYFCTDYCCTDYCCTDYCCTDYCCTDYCCTDYCCTDYCCTDYCCTDYCCTD
YCCTDYCCTDYCCTDYCCTDYCCTDYCCTDYCCTDYCCTDYCCTDYCCTDYCCTDYCCTDYCCTDYCCTD
YCCTDYCCTDYCCTDYCCTDYCCTDYCCTDYCCTDYCCTDYCCTDYCCTDYCCTDYCCTDYCCTDYCCTD
YCCTDYCCTDYCCTDYCCTDYCCTDYCCTDYCCTDYCCTDYCCTDYCCTDYCCTDYCCTDYCCTDYCCTD
YCCTDYCCTDYCCTDYCCTDYCCTDYCCTDYCCTDYCCTDYCCTDYCCTDYCCTDYCCTDYCCTDYCCTD
YCCTDYCCTDYCCTDYCCTDYCCTDYCCTDYCCTDYCCTDYCCTDYCCTDYCCTDYCCTDYCCTDYCCTD
YCCTDYCCTDYCCTDYCCTDYCCTDYCCTDYCCTDYCCTDYCCTDYCCTDYCCTDYCCTDYCCTDYCCTD
YCCTDYCCTDYCCTDYCCTDYCCTDYCCTDYCCTDYCCTDYCCTDYCCTDYCCTDYCCTDYCCTDYCCTD
YCCTDYCCTDYCCTDYCCKRIFVV
>NXN92838.1 KRA43 protein, partial [Rhinopomastus cyanomelas] | 197
CCRPHCCRPHCCRPHCCRPHCCRPHCCRPHCCRPHCCRPHCCRPHCCRPHCCRPHCCRPHCCRPHCCRPH
CCRPHCCRPHCCRPHCCRPHCCRPHCCRPHCCRPHCCRPHCCRPHCCRPHCCRPHCCRPHCCRPHCCRPH
CCRPHCCRPHCCRPHCCRPHCCRPHCCRPHCCRPHCCRPHCCRPHCCRPHCCRPHCC
