# Supplementary material for: Ancient lineages of the keratin-associated protein (KRTAP) genes and their co-option in the evolution of the hair follicle
Source: BMC Ecol Evol. 2023 Mar 20;23:7. doi: 10.1186/s12862-023-02107-z (PMC10029157; doi:10.1186/s12862-023-02107-z)
Supplement: Supplementary file 4 — Additional file 4: Table S2. List of the 19 Phylostrata [file 12862_2023_2107_MOESM4_ESM.docx]

**Additional file 4: Table S2**: List of the 19 Phylostrata:


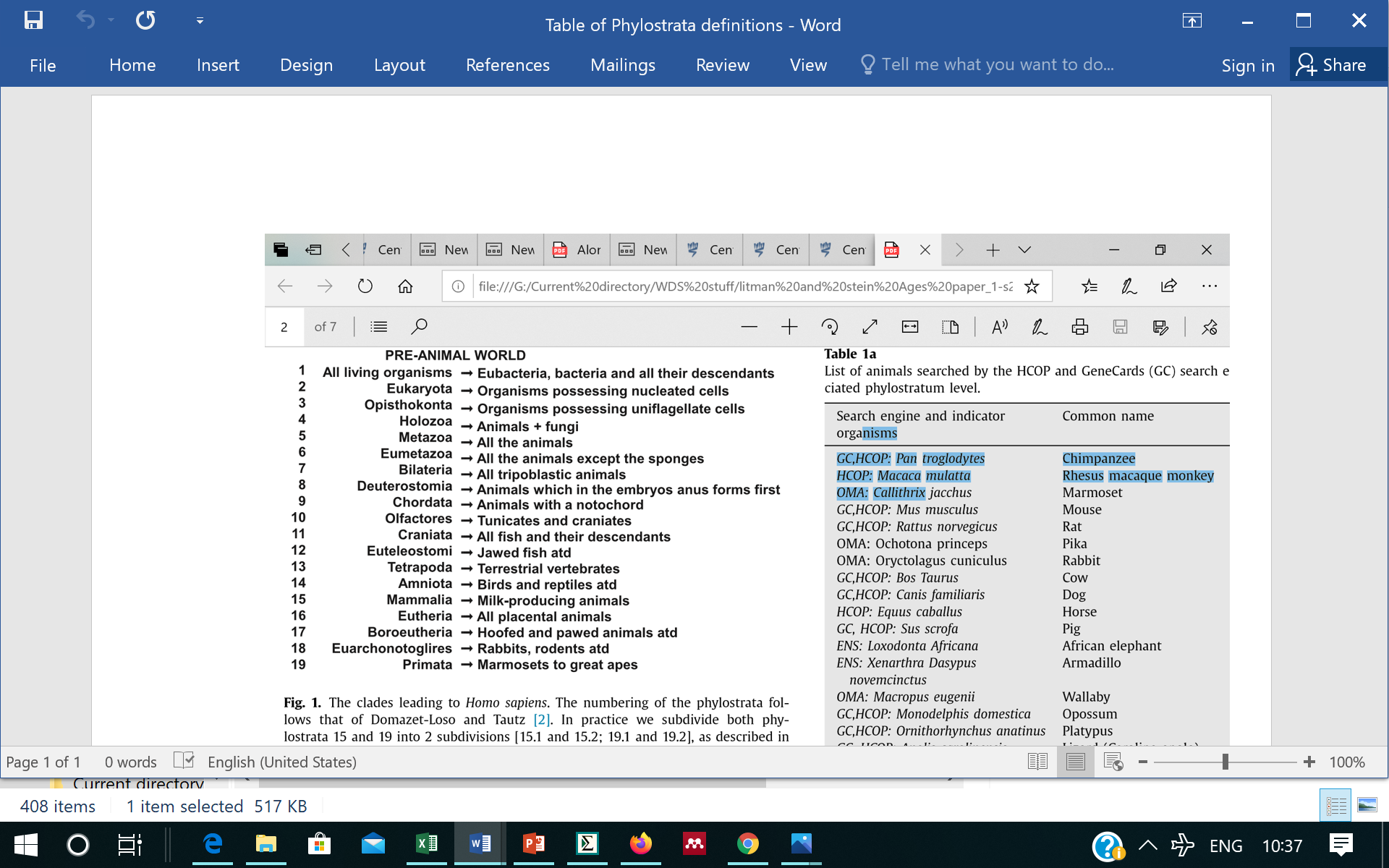


**atd** = and their descendants

List based on Domazets-Loso and Tautz (2010)^14^, as modified by Litman and Stein^12^.
