# Supplementary material for: Ancient lineages of the keratin-associated protein (KRTAP) genes and their co-option in the evolution of the hair follicle
Source: BMC Ecol Evol. 2023 Mar 20;23:7. doi: 10.1186/s12862-023-02107-z (PMC10029157; doi:10.1186/s12862-023-02107-z)
Supplement: Supplementary file 5 — Additional file 5: Table S3. Contenders for the role as ancestral proteins. [file 12862_2023_2107_MOESM5_ESM.docx]

**Additional file 5: Table S3**: Contenders for the role as ancestral proteins

| Gene name | Gene symbol | Count | Phylostratum | Harvest |
| --- | --- | --- | --- | --- |
| Small cysteine and glycine repeat-containing protein 2 | SCGRC2 (KRTAP28-2) | 17 | 17 | Mammal |
| Small cysteine and glycine repeat-containing protein 4 | SCGRC4 (KRTAP28-4) | 17 | 17 | Mammal |
| Small cysteine and glycine repeat-containing protein 6 | SCGRC6 (KRTAP28-6) | 17 | 17 | Mammal |
| SCO-spondin | SSPOP | 16 | 9 | 0 |
| Laminin subunit alpha-5 | LAMA5 | 14 | 7 | 3 |
| Extracellular matrix organizing protein FRAS1 | FRAS1 | 13 | 9 | 0 |
| Cysteine-rich motor neuron 1 protein | CRIM1 | 12 | 7 | 41 |
| Mucin-5B Cervical mucin | MUC5B | 12 | 7 | 0 |
| Neurogenic locus notch homolog protein 1 | NOTCH1 | 12 | 7 | 0 |
| Platelet endothelial aggregation receptor 1 | PEAR1 | 12 | 13 | 10 |
| Teneurin-3 | TENM3 | 12 | 4 | 15 |
| von Willebrand factor | VWF | 12 | 6 | 8 |
| Skin-specific protein 32 [Homo sapiens] | XP32 | 12 | 18 | Mammal |
| Fibrillin-1 | FBN1 | 11 | 3 | 0 |
| IgGFc-binding protein | FCGBP | 11 | 9 | 52 |
| Kielin/chordin-like protein | KCP | 11 | 9 | 34 |
| Late cornified envelope protein 5A | LCE5A | 11 | 19.1 | Mammal |
| Transcriptional repressor NF-X1;Nuclear transcription factor | NFX1 | 11 | 1 | 73 |
| NF-X1-type zinc finger protein NFXL1 | NFXL1 | 11 | 2 | 78 |
| Stabilin-1;Fasciclin | STAB1 | 11 | 12 | 0 |
| Multiple epidermal growth factor-like domains protein 10 | MEGF10 | 10 | 7 | 0 |
| Multiple epidermal growth factor-like domains protein 6 | MEGF6 | 10 | 1 | 0 |
| Mucin-5AC Major airway glycoprotein;Mucin-5 subtype AC | MUC5AC | 10 | 6 | 0 |
| Zonadhesin | ZAN | 10 | 12 | 0 |
| Agrin | AGRN | 9 | 7 | 10 |
| Metallothionein1A | MT1-A | 9 | 7 | 86 |

NOTES:

Count: number of times the gene appeared when the human genome was BLAST-searched by KRTAPs in the upper half of the phylogenetic tree of Fig. 1.

Phylostratum: phylostratum number as defined in Methods

Harvest: number of "keratin-associated protein" hits in the top 1000 sequences when the human genome was BLAST-searched using the gene in question.
